# Two-Dimensional MXene-Loaded Butylphthalide Enhances the Treatment of Alzheimer’s Disease by Inhibiting Ferroptosis and Oxidative Stress

**DOI:** 10.3390/ph19091386

**Published:** 2026-09-01

**Authors:** Yajun Zhou, Bangjian Liu, Hui Wang, Li Cao

**Affiliations:** 1 Department of Neurology, Shanghai Sixth People’s Hospital Affiliated to Shanghai Jiao Tong University School of Medicine Shanghai, 600 Yishan Road, Shanghai 200233, China; 2 Department of Neurology, Seventh People’s Hospital of Shanghai, University of Traditional Chinese Medicine, 358 Datong Road, Pudong, Shanghai 200120, China; 3 Department of Neurology, Tinglin Hospital of Jinshan District of Shanghai, Shanghai 201505, China

**Keywords:** MXene, butylphthalide, Alzheimer’s disease, nanomaterials, neuroprotection

## Abstract

**Introduction**: Alzheimer’s disease (AD) is a chronic progressive neurodegenerative disorder involving ROS and ferroptosis. MXene, a 2D material, functions as both a drug carrier and ROS scavenger. Butylphthalide (NBP), a neuroprotective agent, suffers from poor aqueous solubility and bioavailability. This study synthesized Ti_2_C@BSA-NBP, a novel MXene nanocomposite, to enhance NBP delivery and therapeutic efficacy for AD. **Materials and Methods**: Ti_2_C@BSA-NBP was synthesized via self-assembly and amidation reaction, and characterized by multiple techniques. In vitro, ROS-scavenging capacity, mitochondrial function, and ferroptosis markers were assessed in H_2_O_2_-injured cells. In vivo efficacy was evaluated in an AD mouse model via behavioral, histopathological, and biochemical analyses. Results: The nanocomposite exhibited robust ROS-scavenging activity in vitro, significantly attenuating ROS, restoring mitochondrial function, and reversing ferroptosis markers. In vivo, Ti_2_C@BSA-NBP ameliorated learning/memory deficits, partially repaired neuronal morphology, and suppressed neuroinflammation. Mechanistically, it downregulated ACSL4 and Aβ overexpression while restoring GPX4 inhibition. **Discussion**: Ti_2_C@BSA-NBP exerts synergistic neuroprotection through MXene-mediated ROS clearance and NBP-mediated multi-target regulation, counteracting oxidative stress, ferroptosis, and neuroinflammation. **Conclusions**: Ti_2_C@BSA-NBP is a promising multifunctional nanoplatform integrating antioxidant activity, enhanced drug delivery, and ferroptosis modulation for AD therapy.

## 1. Introduction

Alzheimer’s disease (AD) is a progressive dementia and cognitive decline focused on memory, and thereby, it has been considered as one of the first four diseases that cause death in elderly people [1,2,3]. Its major pathologies comprise amyloid-beta (Aβ) plaques, neurofibrillary tangles by hyperphosphorylated tau protein, neuronal loss, and oxidative stress [4]. Since the population in China is gradually aging and the elderly population continues to rise year by year, the cases of AD also increase [5,6]. AD currently affects at least 50 million people and is anticipated to affect 115 million people worldwide in the year 2050 [7]. Antecedent factors comprise metal elements, infections, and AD-like symptoms [8,9,10,11,12]. At present, no cure exists for AD, but the drugs in use are anti-cholinesterases, β-site secretase, γ-site secretase, A-site stimulate, steady-state tubulins, safe anti-inflammatory drugs, Tau protein kinase, and non-competitive excitatory amino acid receptor (NMDA) blockers [13,14]. While gene therapy and stem cell therapy also offer promising new directions in research [15,16,17], developing new approaches is necessary.

The L-n-butylphthalide (L-NBP) present in the seeds of the Apium graveolens (Chinese celery) exhibits properties of prevention from Alzheimer’s disease (AD). It can reduce Aβ aggregation or its deposition and can break down this protein, decrease neuroinflammation, and increase cerebral blood flow [18,19,20,21,22]. However, the potential advantage of using NBP in the treatment of AD is dampened by its low water solubility and, consequently, low BBB permeability [20,22]. Thus, considerable efforts have been made to search for an appropriate delivery system that enhances the pharmacokinetic behavior and biodistribution of NBP with reduced toxicity.

Over the last few years, nanomedicine has proved to be one of the most exciting and potentially highly effective delivery systems for drugs targeting AD. Nanocarriers can incorporate the drug molecules, shield them from degradation, and deliver in a sustained manner at the requisite site [23,24]. Furthermore, the modification of the surface of nanoparticles with biocompatible substances allows for increasing the permeability of the blood–brain barrier (BBB) and, as a consequence, stabilizes drug delivery to the cerebral tissue and minimizes side effects [25,26,27].

The materials termed MXenes are a category of 2D inorganic materials that consist of layers of transition metal carbides, nitrides or carbonitrides, each of which are only a few atoms thick. The properties of MXenes are highly advantageous for the treatment of AD [28]. Because of their negative charge, they might chelate positive free metal ions [28,29,30,31,32]; the increase in these ions is believed to be related to AD development [8,9,10]. In addition, MXenes are known for their role in the removal of ROS [33,34,35], indicating that 2D MXenes can be used for particular applications in the biomedical field, especially for targeting AD. Additionally, the excellent photothermal conversion efficiency and strong absorption ability in the near-infrared regions of MXenes could lead to immediate tumor cell death; in the context of AD, this property can allow the system to eliminate affected cells in the brain [36,37,38]. Apart from being used in AD treatment, 2D MXenes have shown significant promise in the field of imaging techniques such as fluorescence imaging, photoacoustic imaging, nuclear magnetic resonance imaging, and thermal infrared imaging [37,39]. Furthermore, MXenes are biocompatible, signaling, and water-soluble and have multilayer structures on the nanoscale level that make them useful as attachment points for signal molecules and sensing [40,41,42,43,44]. To enhance the efficacy of MXene-based treatments for AD, some authors have considered the use of these 2D nanocarriers with appropriate particle sizes to deliver desired drugs and create drug-loaded nanoparticles [28]. MXene-based nanocarriers may facilitate nanoparticle transport across the BBB and, with appropriate surface modifications, potentially improve brain-targeting efficiency while minimizing toxic impacts and enhancing therapeutic gains [28]. Consequently, the creation and improvement of MXene-based nanomaterials for human diseases, especially AD, are required.

Beyond their applications in AD therapy, MXenes have recently emerged as promising platforms for neural tissue engineering and regeneration. A comprehensive review by Liao et al. highlighted that MXenes, with their excellent biocompatibility, electrical conductivity, and surface modifiability, serve as good substrates for nerve cell regeneration and nerve reconstruction [45]. Wei et al. demonstrated that 3D conductive Ti3C2Tx MXene-Matrigel hydrogels effectively promoted the proliferation and neuronal differentiation of neural stem cells, providing new strategies for central nervous system injury repair [46]. Tang et al. showed that ultrathin Nb2C MXene enhanced the performance of retinal progenitor cells for retinal regeneration through dual functions of promoting neuronal differentiation and scavenging free radicals [47]. Additionally, Li et al. reported that Ti3C2Tx MXene films promoted the electrophysiological maturation of neural stem cell-derived neurons and the formation of neural networks [48]. Furthermore, Cai et al. developed GelMA-MXene hydrogel nerve conduits with microgrooves for spinal cord injury repair [49]. These studies demonstrate the broad potential of MXene-based materials in the field of neural repair and regeneration, supporting their application in neurodegenerative disease treatment.

Bovine serum albumin (BSA), also known as the fifth component of bovine serum, is a globulin found in bovine blood. It is characterized by 583 amino acid residues and a molecular mass of 69 kilodaltons. Studied in nanomedicine, BSA has received considerable attention in comparison to other biocompatible materials; it has low immunogenicity, a relatively lower cost, and is easily conjugated with drugs [50,51]. BSA nanoparticles have been widely used for drug delivery, gene delivery, and cellular imaging, revealing excellent stability and biocompatibility [52,53,54,55]. Additionally, the use of BSA on nanoparticle surfaces can decrease protein binding and inflammation, thereby increasing the circulation time of nanoparticles in the bloodstream and increasing their performance in drug delivery [56]. Further, BSA nanoparticles have the capability to improve the permeability of the BBB relying on receptor-mediated transcytosis [57,58,59,60,61,62]. These properties of BSA make BSA a good carrier for delivery of NBP in the treatment of AD. In addition, loading NBP with both BSA and MXene flakes may improve the stability and circulation time of the NBP-loaded BSA nanoparticles and afford additional therapeutic effects due to reduced oxidative stress and protection of the nervous system from further degeneration.

Therefore, it is proposed in this present work to develop NBP-incorporated BSA nanoparticles with sustained release, low cytotoxicity, and enhanced permeability to be dispersed onto MXene flakes for potential BBB transport in the management of AD. This newly proposed strategy makes full use of the advantageous properties of both BSA and MXene to enhance the pharmacokinetics, biodistribution, and therapeutic efficacy of NBP for treating AD. This paper is worthy of appreciation of the possible evolution of the new nanomedicine approach toward the treatment of AD with fewer hurdles encountered in the present therapies.

## 2. Results

### 2.1. Synthesis and Characterization of Materials

**Figure 1:**
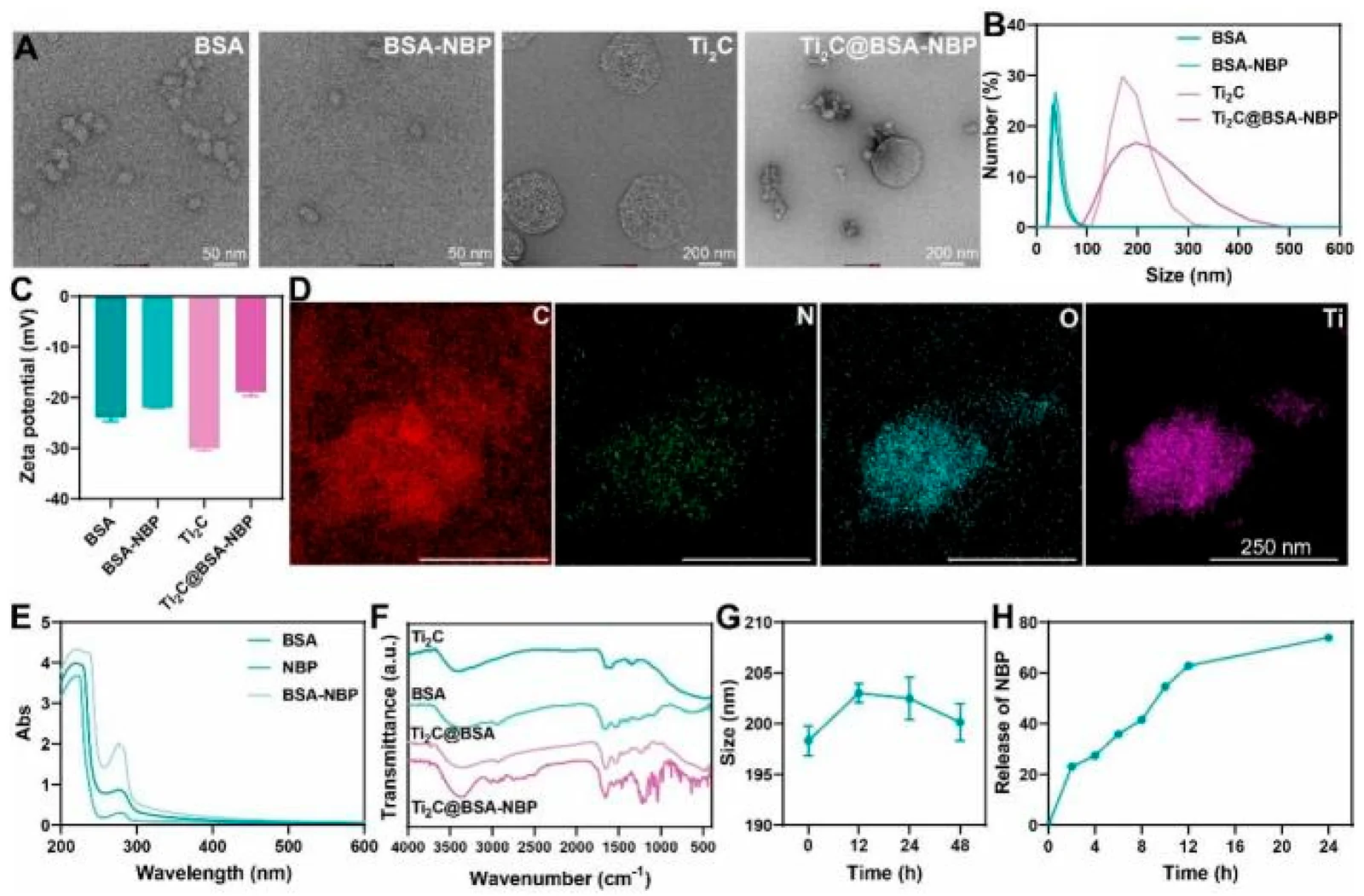
Synthesis and characterization of materials. (**A**) Representative TEM images showing the morphology of BSA, BSA-NBP, and Ti2C@BSA-NBP. Scale bars are indicated on the images. (**B**) Particle size distribution of BSA, BSA-NBP, and Ti2C@BSA-NBP measured by DLS. (**C**) Zeta potential of BSA, BSA-NBP, and Ti2C@BSA-NBP. (**D**) Elemental mapping of Ti2C@BSA-NBP, confirming the distribution of Ti elements. (**E**) UV–visible absorption spectra of BSA, NBP, and BSA-NBP. (**F**) FTIR spectra of BSA, Ti2C@BSA, and Ti2C@BSA-NBP. (**G**) Stability of Ti2C@BSA-NBP in aqueous solution over 24 h, measured by DLS. (**H**) Cumulative drug release profile of BSA-NBP over 24 h. Data are expressed as mean ± SD (*n* = 3).

BSA and BSA-NBP NPS were synthesized by self-assembly method, and then BSA-NBP NPs was grafted on flaky MXene by amidation reaction to prepare Ti2C@BSA-NBP. TEM images of BSA, BSA-NBP, and Ti2C@BSA-NBP (A) are shown in Figure 1A. TEM analysis revealed BSA and BSA-NBP to have similar spherical structures with rough surfaces, which means that morphology, size, and shape did not change significantly upon loading the drug. Alternatively, BSA-NBP incorporation into Ti2C enhanced the formation of a flaky morphology, which was also persistent, indicating that nanoparticles did attach to the surface of Ti2C. The particle size distribution analysis demonstrated in Figure 1B showed that both BSA and BSA-NBP had a particle size of approximately 40 nm (PDI = 0.21 ± 0.03 and 0.24 ± 0.04, respectively), but Ti2C had a larger particle size of about 200 nm (PDI = 0.26 ± 0.05) (*n* = 3, mean ± SD). Following the grafting of BSA-NBP to Ti2C, the particle size distribution of the resultant Ti2C@BSA-NBP was broader than that of Ti2C alone, suggesting that the grafting resulted in an increase in size variability as a result of the addition of BSA-NBP. The synthesized nanomaterials’ zeta potential measurements are illustrated in Figure 1C. The potential of BSA and its Ni(II) coordination complex BSA-NBP were recorded at −23.91 mV and −22.06 mV, respectively, thus showing a slight increase in positivity after the incorporation of NBP. Conversely, a more negative potential of −30.11 mV was observed for Ti2C, which changed to −18.92 mV after grafting BSA-NBP. This potential reduction confirmed the successful BSA-NBP attachment to the Ti2C surface. Figure 1D represents the elemental mapping, thereby confirming the Ti distribution in the composite Ti2C@BSA-NBP, as shown in Figure 1D. Ti mapping revealed an even distribution of the element to reinforce the suggestion that the particles were effectively immobilized. UV–visible absorption spectra for BSA, NBP, and BSA-NBP are shown in Figure 1E. Importantly, the UV spectrum of BSA-NBP exhibited strong absorption peaks of NBP that were usually present (275–280 nm), indicating that we had created a composite compound between BSA and NBP. This synthesis is further confirmed by the infrared (IR) spectra in Figure 1F. In the Ti2C spectrum, a stretching vibration peak at 1656 cm^−1^ was observed for C=O bonds. In BSA, the peaks around 1535 cm^−1^ and 1394 cm^−1^ were attributable to C-N stretching and N-H bending vibrations, respectively, corresponding to the characteristic amide II band [63]. In the IR spectrum of Ti2C@BSA-NBP, these peaks were shifted and intensified, with the peak at 1656 cm^−1^ corresponding to the amide I band (C=O stretching of amide bonds) and the peak at 1535 cm^−1^ corresponding to the amide II band (N-H bending and C-N stretching), collectively indicating the formation of new amide bonds through the amidation reaction between BSA-NBP and Ti2C [64]. This spectroscopic evidence is consistent with FTIR-based confirmation of amide bond formation widely used in protein-nanomaterial conjugation studies [65]. The stability analysis of Ti2C@BSA-NBP is illustrated in Figure 1G, where the hydrodynamic diameter was monitored in PBS at room temperature over 48 h (0, 12, 24, and 48 h). The particle size remained stable with changes of less than 10% throughout the observation period, confirming the colloidal stability of the composite in physiological buffer conditions. The release profile of BSA-NBP is shown in Figure 1H, where about 73% of the drug was released in 24 h. This result indicated that the synthesis enabled an appropriate control release mechanism for the entering drug. X-ray diffraction (XRD) analysis data in Figure S1 showed the XRD pattern of Ti2C and Ti2C@BSA-NBP. Results showed that the synthesis of the Ti2C@BSA-NBP composite largely retained the crystalline structure of Ti2C, for example, signifying that Ti2C was not substantially altered during the grafting process. Finally, we tested the rate of BSA–NBP loading and encapsulation into NBP vesicles shown in Figure S2. Loaded rate and encapsulation rate were found to be 12.98% and 37.28%, respectively. The efficiency of the synthesis process and potential for practical applications of this material in drug delivery systems were quantitatively measured.

### 2.2. Ti2C with Enzyme-Mimicking Activity as a Scavenger of Reactive Oxygen Species

**Figure 2:**
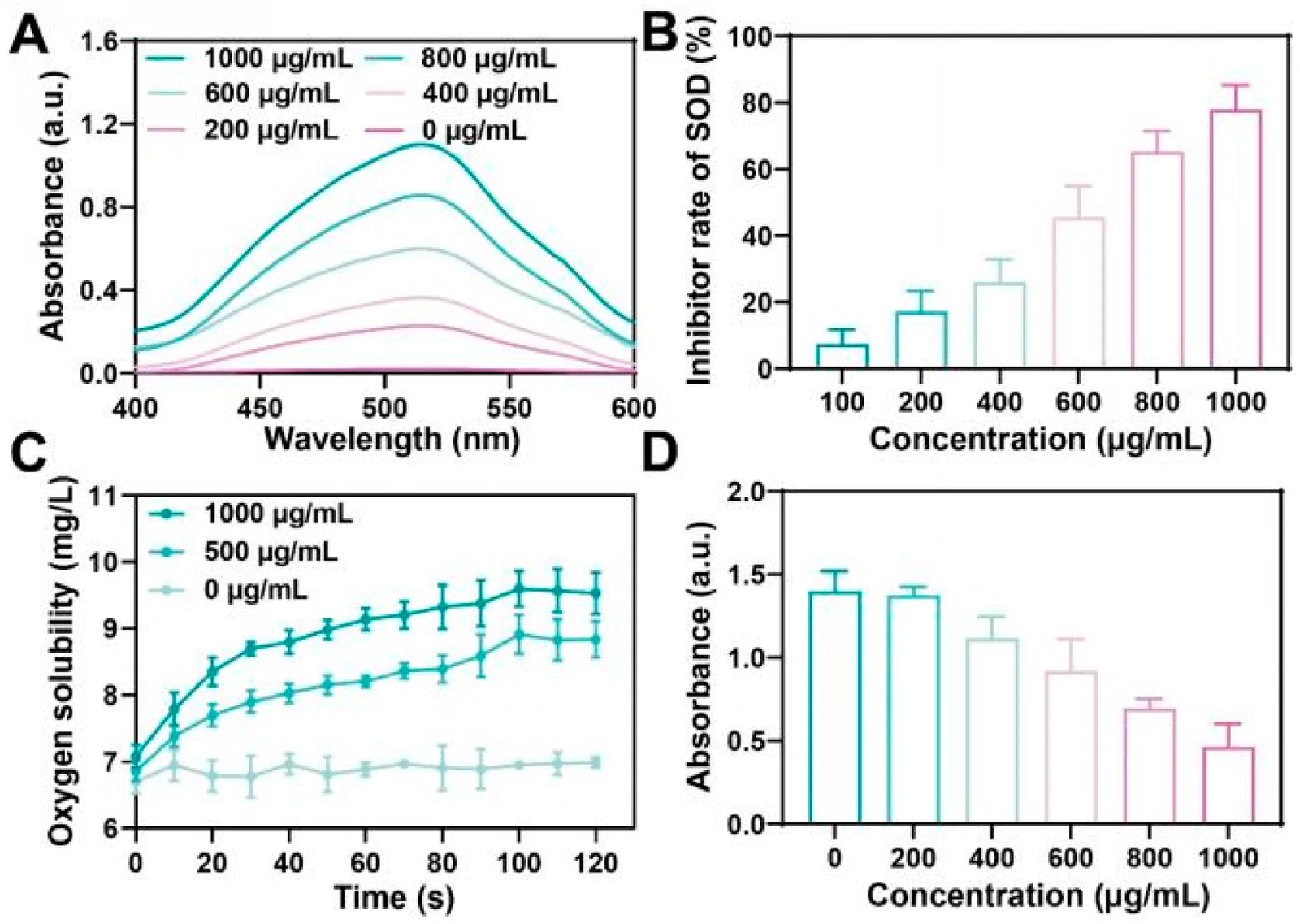
Enzyme-mimicking activity of Ti_2_C for scavenging reactive oxygen species. (**A**) Total antioxidant capacity of Ti_2_C. (**B**) SOD-mimicking activity of Ti_2_C. (**C**) CAT-mimicking activity of Ti_2_C. (**D**) GPX-mimicking activity of Ti_2_C.

To assess the ROS-quenching, enzymatic nature of Ti2C, the TAC method was used to determine the antioxidant potentiality. The observed antioxidant activity of Ti2C alkaline material enhances with concentration, as revealed in Figure 2A. Next, the SOD, CAT, and GPx enzyme-mimicking activity of Ti_2_C were measured. The main component of ROS is O_2_ · -. SOD helps convert toxic O2 ·- into H_2_O_2_ and O_2_, enabling cells to cope with high levels of oxidative stress.

SOD activity of Ti2C was determined by using WST-1, a reagent whose product formazan at 450nm is inhibited by O2 ·-. In this way, we found that the presence of Ti2C effectively reduced the O2·- and suppressed the formation of formazan. With the enhancement of Ti2C concentration, formazan formation was reduced, showing that Ti2C possesses SOD-like activity (Figure 2B). H2O2, the agent we utilize for our oxidative stress test, is broken down by CAT to H2O + O2, so it cannot build up. Like CAT, Ti2C showed activity in producing O2 from H2O2. When Ti2C was reacted with H2O2, bubbles containing O2 were found to be formed, and frequently, the number of bubbles of O2 over time (Figure 2C) demonstrated that Ti2C possesses CAT activity.

H_2_O_2_ concentration is reduced in cells by GPx. For this purpose, GPx utilizes intracellular GSH by first reducing it to an H_2_O_2_ reduction to H_2_O, in the process oxidizing glutathione (GSSG), and then glutathione reductase (GR) and NADPH recycle GSH from GSSG. In order to evaluate the GPx-like activity of Ti_2_C, we used a GR-coupled method to determine the activity of the enzyme. This method entails the changes in absorbencies at a wavelength of 340 nm and measures the rate of reduction in NADPH. The overall trend of the NADPH absorbance to decline with increasing v concentration is presented in Figure 2D. Finally, Ti2C displays enzyme-like activity and is shown to be a very effective ROS scavenger.

### 2.3. Ti_2_C@BSA-NBP Can Reduce ROS and Improve Mitochondrial Function

**Figure 3:**
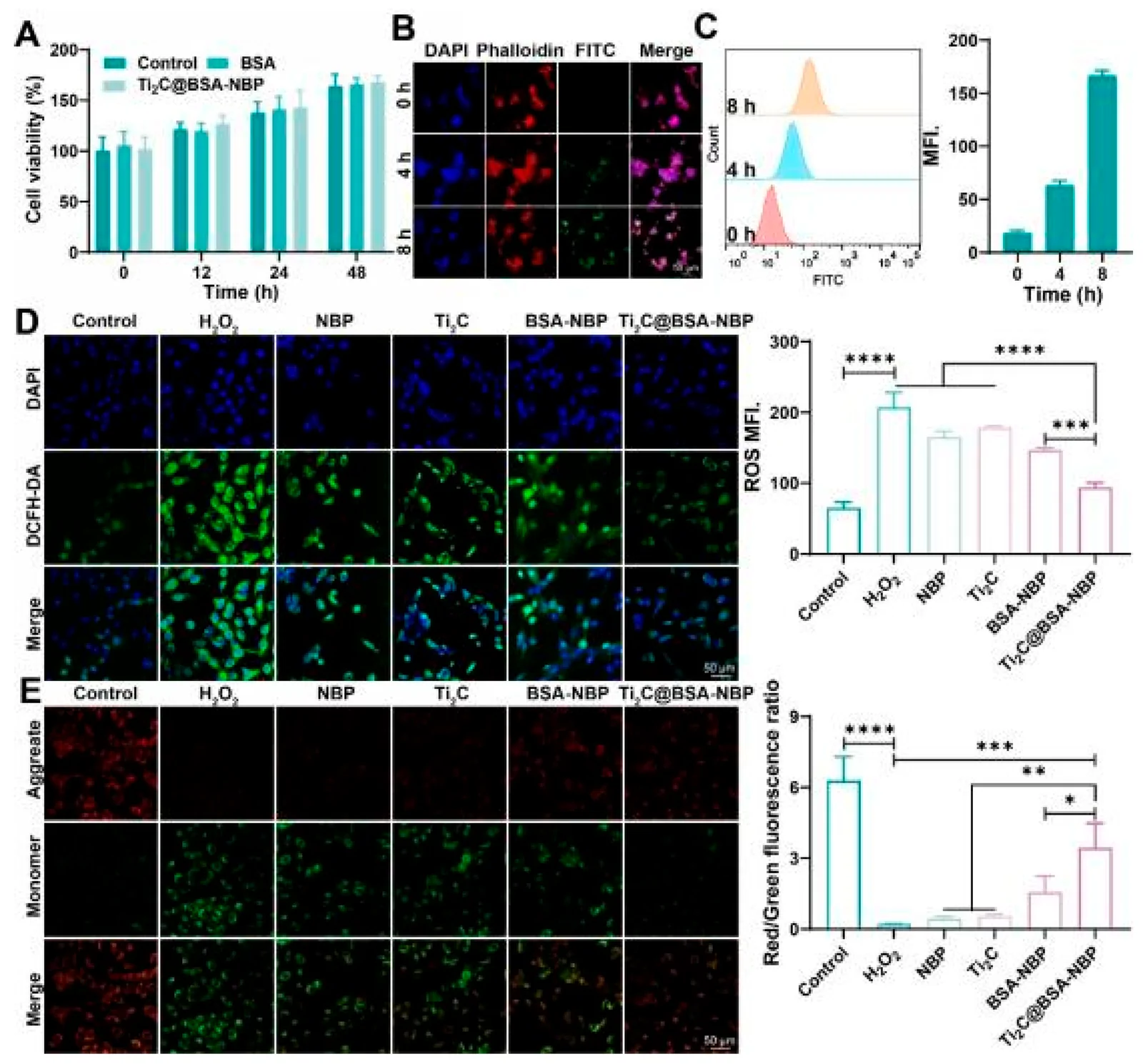
Ti2C@BSA-NBP can reduce ROS and improve mitochondrial function. (**A**) CCK8. (**B**,**C**) Phagocytosis. (**D**) ROS levels. (**E**) Mitochondrial membrane potential detected by JC-1 staining. *n* = 3, * represents *p* < 0.05, ** represents *p* < 0.01, *** represents *p* < 0.001, **** represents *p* < 0.0001.

The results of the CCK8 assay analysis shown in Figure 3A showed that the materials tested did not significantly affect cell viability, and the materials are biologically safe. Endocytosis of debris by cells (Figure 3B,C) was observed to be an increasing function of time. For the ROS levels (Figure 3D), the results showed that, compared with the Control group, the ROS level of the H2O2 model group rose significantly, and the ROS level reduced after the incorporation of several materials. The biggest contrast was noticed with the Ti2C@BSA-NBP, implying that Ti2C@BSA-NBP advanced incredible antioxidant potential. Last, the outcome of JC-1 fluorescent staining demonstrated that in the H2O2 model group, the fluorescence density of JC-1 intensely declined, suggesting that the mitochondrial membrane potential significantly decreased, while upon the addition of various materials, the fluorescence density of JC-1 increased (Figure 3E). As expected, the Ti2C@BSA-NBP group depicted the highest values of difference, thus demonstrating that Ti2C@BSA-NBP can improve mitigation functionality.

### 2.4. Ti2C@BSA-NBP Can Alleviate H2O2-Induced Ferroptosis

**Figure 4:**
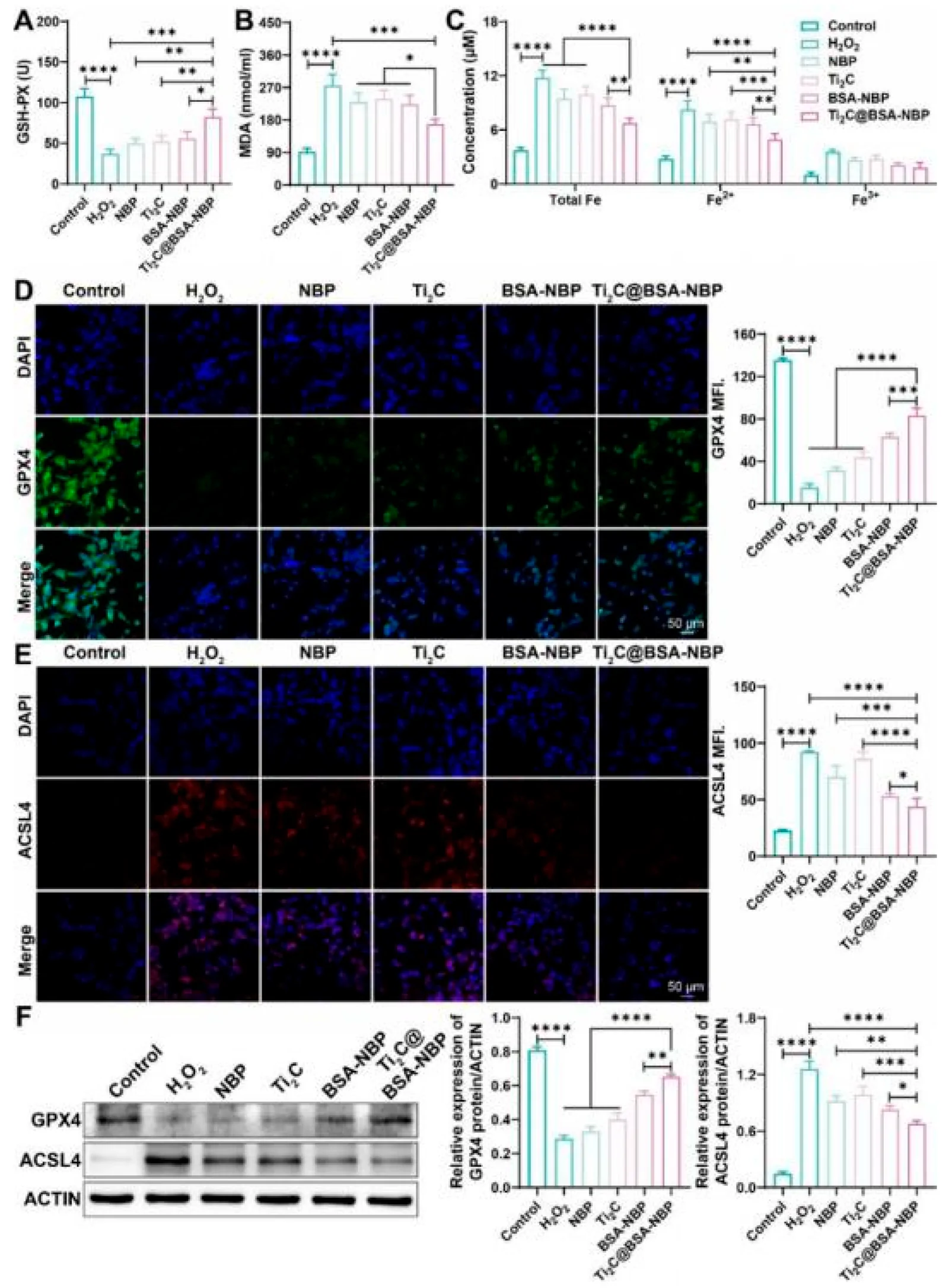
Ti2C@BSA-NBP can alleviate H2O2-induced ferroptosis. (**A**) GSH-PX levels in different treatment groups. (**B**) MDA levels in different treatment groups. (**C**) Iron ion levels (total iron and Fe^2+^) in different treatment groups. (**D**) Representative immunofluorescence images of GPX4 expression. (**E**) Representative immunofluorescence images of ACSL4 expression. (**F**) Western blot analysis of GPX4 and ACSL4 protein expression, with quantitative densitometry. Data are expressed as mean ± SD (*n* = 3 independent biological replicates, each with 3 technical replicates). Statistical analysis was performed using one-way ANOVA followed by Tukey’s post hoc test. * represents *p* < 0.05, ** represents *p* < 0.01, *** represents *p* < 0.001, **** represents *p* < 0.0001.

A marked decrease in GSH-PX level was observed in the H2O2 model group compared to the Control group, while the levels of MDA, total iron ions, and Fe^2+^ significantly increased when compared to the Control group (Figure 4A–C). After treatment, GSH-PX levels were upregulated, MDA, total iron ions, and Fe^2+^ were downregulated, and the differences were most significant in the Ti2C@BSA-NBP group. Immunofluorescence staining (Figure 4D,E) and Western Blot (Figure 4F) were used to determine GPX4/ACSL4 and found that GPX4 was significantly lower in the H2O2 model group than in the Control group, while ACSL4 expression was significantly higher in the H2O2 model group as compared to the control group. After adding different materials, the expression of GPX4 was upregulated, while the expression of ACSL4 was downregulated; the change was the largest in the Ti2C@BSA-NBP group, suggesting that Ti2C@BSA-NBP can inhibit H2O2-induced ferroptosis. ACSL4 (Acyl-CoA Synthetase Long Chain Family Member 4) is a key enzyme that incorporates polyunsaturated fatty acids (PUFAs) into phospholipids, making cells more susceptible to lipid peroxidation and ferroptosis. Upregulation of ACSL4 has been closely associated with ferroptosis activation in neurodegenerative diseases, including Alzheimer’s disease.

**Figure 5:**
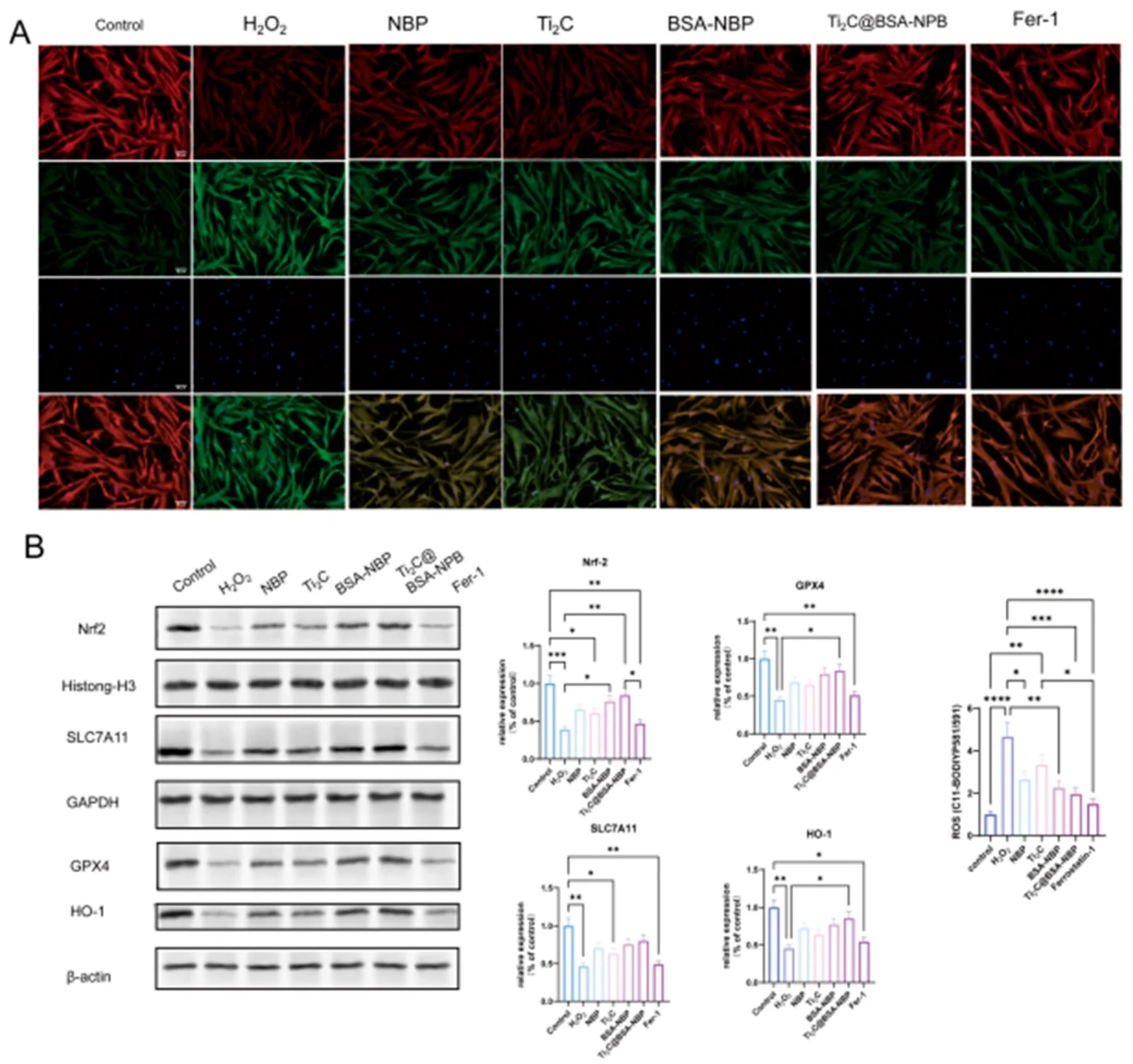
Mechanistic validation of Ti2C@BSA-NBP-mediated ferroptosis inhibition in H2O2-injured PC12 cells. (**A**) Representative C11-BODIPY 581/591 staining images showing lipid ROS levels (green: oxidized; red: reduced; blue: DAPI) and quantitative analysis of the green/red fluorescence ratio. (**B**) Western blot analysis of nuclear Nrf2, HO-1, SLC7A11, and GPX4 protein expression, with Histone H3 and β-actin as loading controls, and corresponding densitometric quantification. Data are expressed as mean ± SD (*n* = 6 independent biological replicates). Statistical analysis was performed using one-way ANOVA followed by Tukey’s post hoc test. * *p* < 0.05, ** *p* < 0.01, *** *p* < 0.001, **** *p* < 0.0001.

To further elucidate the molecular mechanism underlying the anti-ferroptotic activity of Ti2C@BSA-NBP, we examined the Nrf2/HO-1/SLC7A11/GPX4 signaling axis by Western blotting and directly monitored lipid peroxidation using the C11-BODIPY 581/591 probe (Figure 5). H2O2 treatment markedly elevated lipid ROS levels, as evidenced by a green/red fluorescence ratio of 4.658 ± 1.851 relative to the Control group (1.000 ± 0.397). Ti2C@BSA-NBP treatment significantly reduced lipid ROS accumulation to 1.952 ± 0.776, a level comparable to the Ferrostatin-1 positive control (1.494 ± 0.594). Western blot analysis of nuclear/cytoplasmic fractions revealed that H2O2 significantly decreased nuclear Nrf2 and its downstream targets HO-1, SLC7A11, and GPX4. Ti2C@BSA-NBP treatment restored these protective proteins to 0.843 ± 0.153 (Nrf2), 0.855 ± 0.155 (HO-1), 0.800 ± 0.145 (SLC7A11), and 0.838 ± 0.152 (GPX4) of Control levels, representing the strongest recovery among all treatment groups. These results confirm that Ti2C@BSA-NBP alleviates H2O2-induced ferroptosis by activating the Nrf2/HO-1/SLC7A11/GPX4 axis and suppressing lipid peroxidation.

### 2.5. In Vivo Imaging of Ti2C and Ti2C@BSA Labeled with cy5

The materials Ti2C and Ti2C@BSA (the precursor materials of Ti2C@BSA-NBP) labeled with Cy5 were injected into mice through the tail vein, and in vivo imaging was performed at 4 h and 12 h to observe the distribution of the materials in the mice (Figure S3). The results showed that both Ti2C and Ti2C@BSA showed brain accumulation, with Ti2C@BSA exhibiting relatively higher brain enrichment (Figure S3). It should be noted that these imaging experiments were conducted using Ti2C and Ti2C@BSA precursors rather than the final Ti2C@BSA-NBP composite; nevertheless, the BSA-mediated brain enrichment trend is expected to be preserved in the composite formulation. BSA is bovine serum albumin, which has good biocompatibility and low immunogenicity (Figure S3). It can encapsulate drugs to form nanoparticles with a suitable particle size and potentially improve the permeability of the blood–brain barrier; MXene (Ti2C) is a new type of 2D nanomaterial with good biocompatibility (Figure S3). It can play a role in simulating enzyme-mimicking activity, actively clearing ROS in the lesion site, reducing inflammation, inhibiting oxidative stress, and protecting the nervous system after drug release (Figure S3). BSA is loaded with the drug NBP to form nanoparticles, providing sustained release, toxicity reduction, and enhanced penetration (Figure S3). The formed nanoparticles are further loaded on the sheet-like MXene and administered intravenously (Figure S3). The drug is expected to be delivered across the blood–brain barrier, which may contribute to the treatment of AD.

### 2.6. Ti2C@BSA-NBP Can Rescue Hippocampal Neuron Damage in AD Mice

**Figure 6:**
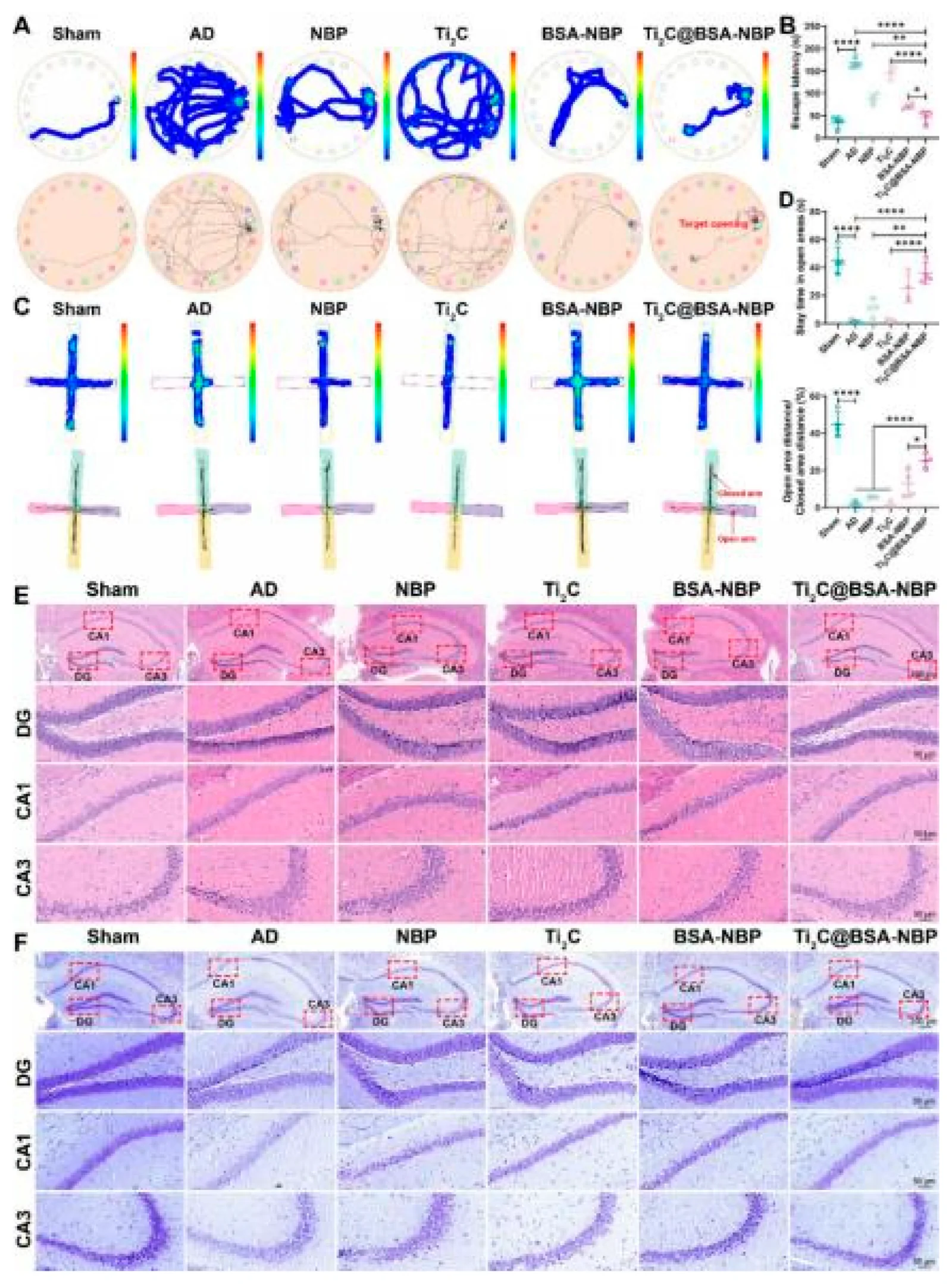
Ti2C@BSA-NBP can rescue hippocampal neuron damage in AD mice. (**A**–**D**) Behavioral tests: (**A**,**B**) Barnes maze test for spatial learning and memory, (**C**,**D**) Elevated plus maze test for anxiety-like behavior. (**E**) H&E staining of hippocampal sections (left: representative images; right: quantitative analysis of neuronal density). (**F**) Nissl staining of hippocampal sections (representative images). Quantitative analysis of optical density in DG, CA1, and CA3 is shown in Supplementary Figure S5. Scale bars are indicated on the images. Data are expressed as Mean ± SD (*n* = 3–4 mice per group). Statistical analysis was performed using one-way ANOVA followed by Tukey’s post hoc test. * represents *p* < 0.05, ** represents *p* < 0.01, **** represents *p* < 0.0001.

In the present study, mouse behavioral testing was carried out using the Barnes maze (Figure 6A,B) and elevated cross (Figure 6C,D) for testing cognitive and memory performance in AD mice. This study showed that significantly increased latency to find the target hole was observed in the Barnes maze of the AD model group of mice in comparison to the Sham group. The time taken by mice to locate the target hole was reduced with the addition of various materials and was most clearly seen in the Ti2C@BSA-NBP group. Likewise, commonly, where the cross results were also higher, it has been illustrated that in comparison to the Sham group, the time interval mice spent in the open arm area and the movement distance ratio between the open field area and the closed field area of the AD model group were significantly minimized. After introducing different types of nanomaterials, the time that mice spent on the open arm significantly decreased, and simultaneously, the movement distance ratio between the open area and the closed area also significantly increased. The present work compared with the AD model group showed the most significant differences in the Ti2C@BSA-NBP group; thus, it can be concluded that Ti2C@BSA-NBP could alleviate the learning and memory deficits of AD mice. Hippocampal neurons in AD mice were examined histologically using H&E staining (Figure 6E) and Nissl staining (Figure 6F). The Sham group was characterized by tightly arranged neurons, large and clear nuclei, less cytoplasm, and perfectly normal architecture. On the other hand, the neurons of the hippocampus in the AD model group were disorganized, had fewer and larger neurons and neuronal nuclei with pyknotic and irregular shapes, and no lytic cells in contrast to the control. Some restoration of neuron shape and form was noticed after the application of various materials. However, significant differences were seen in the Ti2C@BSA-NBP group. The remaining part of the cortex from the AD model group had significantly fewer Nissl bodies than the cortex of the Sham group. The count of the stained Nissl bodies after adding different materials and the differences were prominent in the Ti2C@BSA-NBP group. Quantitative analysis of Nissl staining (optical density, normalized to Sham = 100%) revealed that the optical density in the hippocampal DG, CA1, and CA3 regions was significantly decreased in the AD group (73.8%, 62.9%, and 54.8% of Sham, respectively, all *p* < 0.01 vs. Sham), indicating substantial neuronal loss and reduced metabolic activity. Treatment with Ti2C@BSA-NBP markedly attenuated this reduction, restoring optical density to 88.1%, 74.3%, and 81.0% of Sham levels in DG, CA1, and CA3, respectively (*p* < 0.05 vs. AD). Notably, Ti2C@BSA-NBP demonstrated superior neuroprotective effects compared with free NBP or Ti2C alone, supporting the synergistic benefit of the nano-delivery system (Supplementary Figure S5). These results collectively indicate that Ti2C@BSA-NBP can reverse hippocampal neuronal injury in AD mice.

**Figure 7:**
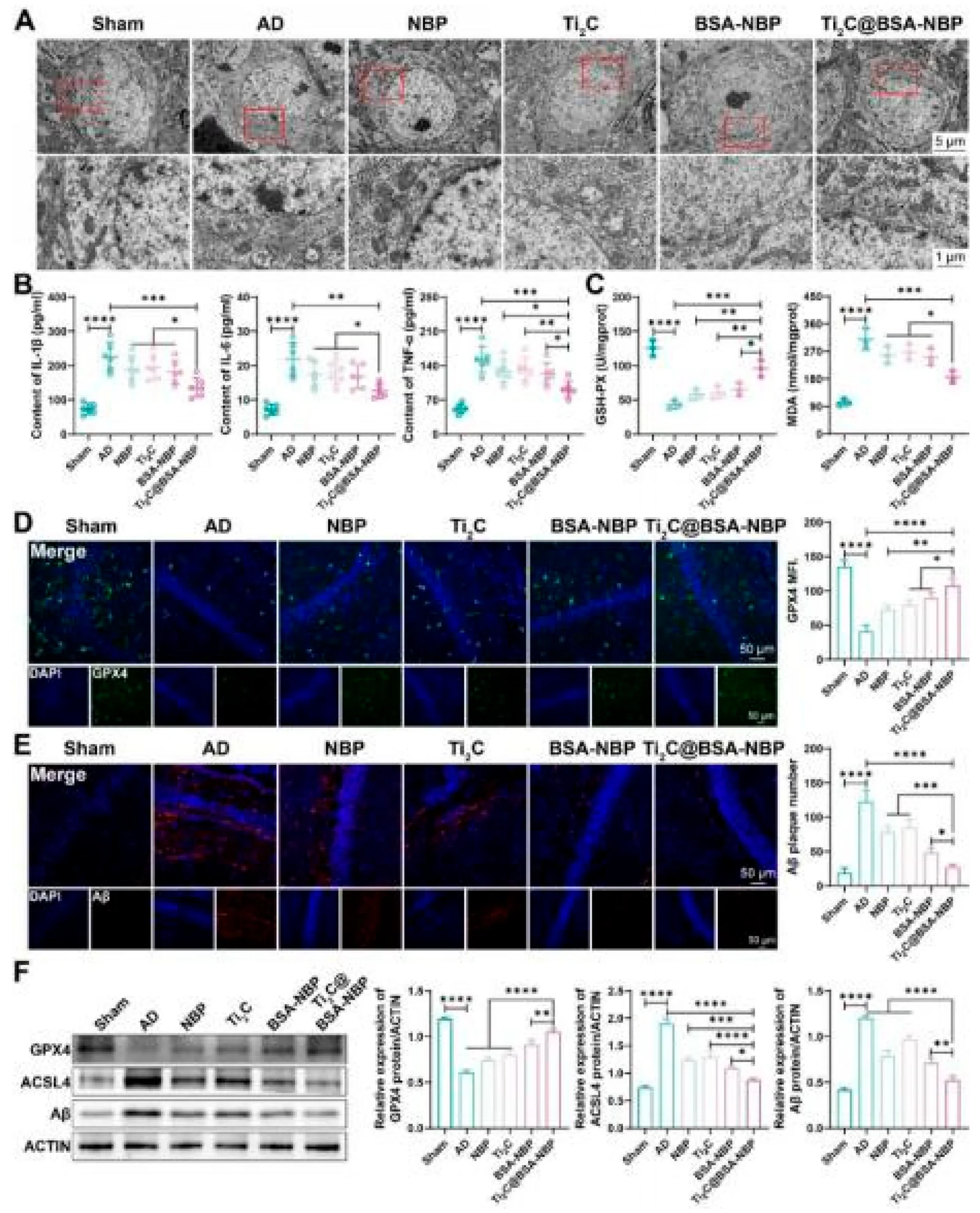
Ti2C@BSA-NBP alleviates neuronal ferroptosis in the brain of AD mice. (**A**) Representative TEM micrographs of hippocampal mitochondria. Quantitative morphometric analysis (mitochondrial area, aspect ratio, circularity, and cristae density) is shown in Supplementary Figure S5. (**B**) IL-1β, IL-6, and TNF-α levels were detected with ELISA. (**C**) GSH-PX and MDA levels. (**D**) GPX4 expression was assessed by immunofluorescence staining. (**E**) Immunofluorescence staining to detect Aβ expression. (**F**) Western blot detects the expression of GPX4, ACSL4 and Aβ. *n* = 3–6, * represents *p* < 0.05, ** represents *p* < 0.01, *** represents *p* < 0.001, **** represents *p* < 0.0001.

As seen in the TEM analysis presented in Figure 7A, the mitochondria of the brain neurons of the Sham group mice were also fully developed. Compared with the Sham group, the brain neurons of mice treated by D-gal in this study formed the AD model group. The mitochondrial damage was significant, and the morphology structure of mitochondria were destroyed, swollen and even ruptured. Longitudinal mitochondria were annulled, and mitochondria of neurons were recovered to some extent after the addition of various materials. Among all the study groups, the Ti2C@BSA-NBP had the maximum impression. Quantitative morphometric analysis of mitochondria revealed that AD mice exhibited significantly increased mitochondrial area (266% of Sham, indicating swelling), decreased aspect ratio (65% of Sham, indicating loss of elongated morphology), and a higher proportion of swollen mitochondria (42% vs. 0% in Sham). Ti2C@BSA-NBP treatment partially restored mitochondrial morphology, with improved cristae density (0.279 vs. 0.250 in AD) and reduced percentage of swollen mitochondria (30% vs. 42% in AD) (Supplementary Figure S5). In ELISA (Figure 7B), it was found that, as compared to the Sham group, the concentration of IL-1β, IL-6, and TNF-α in mice in the AD model group was elevated. Following the addition of various materials, IL-1β, IL-6, and TNF-α levels were attenuated, and the IL-1β, IL-6, and TNF-α of each group exhibited the largest difference in the Ti2C@BSA-NBP group. In mice in the Sham group, as compared with those in the AD model group, the GSH-PX level was decreased, and the value of MDA (Figure 7C) was increased. The results showed that after uniting different materials, the GSH-PX level went high, and the MDA level went low. The Ti2C@BSA-NBP group was found to have the most different expressions from the other groups. Immunofluorescence staining (Figure 7D,E) and Western blot (Figure 7F) results of mice showed that the level of GPX4 in mice in the AD model group was downregulated, and the levels of ACSL4 and Aβ were upregulated, as compared with those in the Sham group. Upon incorporating different materials, the GPX4 expression enhanced, whereas the ACSL4 and Aβ expression reduced notably, especially in the Ti2C@BSA-NBP group. This suggests that Ti2C@BSA-NBP can combat neuronal ferroptosis in the brain of AD mice.

### 2.7. Safety Analysis of the Nanomaterials

The results in Figure S4 in the subsection “Biocompatibility of Ti2C for scavenging reactive oxygen species” show that the H&E staining of histological sections of the heart, liver, spleen, lung, and kidney did not show the presence of conspicuous morphological alterations. These outcomes suggest that the materials investigated in each group lacked cytotoxicity and thus showed moderate biocompatibility, a desirable property for scavenging ROS. As can be evidenced in Figure S4, subpanels representing different organs, the findings are in concordance with previous works and confirm that the studied materials are safe for further investigation and application in various biomedical fields.

## 3. Discussion

For the synthesis of this novel composite in the present study, NBP-loaded BSA nanoparticles were conjugated onto the Ti2C MXene surface through an amidation process. The formation of the composite was also characterized by TEM, zeta potential, elemental mapping, UV–visible absorption spectra, and IR spectrum. Specifically, the FTIR analysis revealed characteristic shifts in the amide I (1656 cm^−1^) and amide II (1535 cm^−1^) bands, providing spectroscopic evidence for amide bond formation between BSA-NBP and Ti2C [64,65]. While X-ray photoelectron spectroscopy (XPS) would offer complementary elemental-level confirmation of the covalent linkage, the convergence of multiple independent characterization techniques—including zeta potential changes, elemental mapping, and spectroscopic evidence—collectively supports the successful formation of the Ti2C@BSA-NBP composite through amidation. Our findings corroborate the previous work of other authors on drug delivery using MXene-based composites [66,67]. Further, the synthesized Ti2C@BSA-NBP composite exhibited high drug encapsulation efficiency (37.28%) and loading capacity (12.98%), which are comparable to or better than other nanoformulations reported for NBP delivery. Previous studies on NBP nanoformulations have reported encapsulation efficiencies ranging from 15 to 35% [21,22], suggesting our composite has competitive drug loading performance. Notably, the same Ti2C@BSA nanocarrier platform has been recently employed to deliver isoquercetin (ISO) for ischemic stroke treatment [68], where comparable particle size distribution, zeta potential profiles, and FTIR characterization patterns were observed. This consistency in physicochemical properties across different drug-loaded formulations further validates the reproducibility and versatility of the Ti2C@BSA-based drug delivery system for neurological disease applications. The physicochemical stability of the applied Ti2C@BSA-NBP composite, as well as its controlled release characteristics under a physiological environment, are crucial factors for drug delivery applications. The sustained release profile observed (approximately 73% drug release within 24 h) is consistent with the desired controlled release behavior for chronic neurodegenerative disease treatment.

### 3.1. Mechanism of Antioxidant Action

The results of the antioxidant abilities of Ti2C demonstrated that the ability to eliminate ROS through SOD-like, CAT-like, and GPx-like functions was possible because of the 2D MXene inherent antioxidant properties, as reported in earlier studies [28,33,34,35]. This multi-enzyme mimicking activity distinguishes Ti2C from conventional antioxidant materials, which typically exhibit single-mechanism ROS scavenging. The data from the in vitro ROS quantitative assays and mitochondrial membrane potential quantitative assays provide evidence for the antioxidant activity of the compound, as Ti2C@BSA-NBP was able to reduce ROS levels and ameliorate MMP dysfunction, which are hallmarks of AD pathology [69]. These findings suggest that, by reducing biomarkers of oxidative stress and mitochondrial dysfunction, Ti2C@BSA-NBP could help improve the treatment of AD and slow disease progression.

### 3.2. Anti-Ferroptotic Effects

The results from the experimental data highlight that Ti2C@BSA-NBP has the potential to attenuate ferroptosis-associated markers, a significant pathological process associated with AD. This is evidenced by the Ti2C@BSA-NBP-instigated increased level of GSH-PX activity, decreased levels of MDA, and reduced iron ion concentration in H2O2-injured cells. Additionally, the DCFH-DA assay (Figure 3D) demonstrated that Ti2C@BSA-NBP significantly reduced total intracellular ROS levels, and Western blot analysis confirmed the modulation of key ferroptosis regulators (GPX4 and ACSL4), providing multi-dimensional evidence for the anti-ferroptotic activity of the composite. GSH-PX represents a powerful antioxidant enzyme that neutralizes extra peroxides, while MDA is an index of lipid peroxidation; moreover, iron ions are strong stimulators of ferroptosis [70]. Our results corroborate other works pointing to oxidative stress and iron-dependent lipid peroxidation in AD pathogenesis [71]. The increase in GSH-PX activities and decrease in MDA and Fe2+ contents in Ti2C@BSA-NBP-treated cells imply that this NBP-loaded MXene formulation could ameliorate oxidative stress and iron overload and interrupt ferroptosis of neurons. Furthermore, the study indicated that Ti2C@BSA-NBP modulates two key ferroptosis proteins (GPX4 and ACSL4). The attachment of Ti2C@BSA-NBP enhances GPX4, a critical enzyme responsible for the detoxification of lipid peroxides, thus allowing the possibility of additional augmentation of the antioxidant status of cells and prevention of lipid peroxidation [65,69]. On the other hand, the downregulation of ACSL4, an enzyme that actively synthesizes polyunsaturated fatty acids, which are easily peroxidizable, also corroborates the anti-ferroptotic effect of Ti2C@BSA-NBP [72].

### 3.3. Neuroprotective Effects and Cognitive Improvement

In this study, the Ti2C@BSA-NBP nanocomposite demonstrated neuroprotective effects that reversed neuronal loss and cognitive and memory dysfunction observed in AD mice. Improved cognitive and memory in mice, evidenced by lower latency in the Barnes maze and increased time spent in the open arms of the elevated plus maze, corroborate the potential therapeutic application of Ti2C@BSA-NBP in AD. These results corroborate previous studies on cognitive and memory impairment in AD and the ameliorative effects of NBP on these effects [21]. The results of H&E and Nissl staining support the assertion that Ti2C@BSA-NBP can reverse the morphology of hippocampal neurons in AD mice, as evidenced in other studies using nanomaterials and compounds to improve neuronal morphology [73]. Furthermore, our study supports observations made by other researchers that NBP has the potential to inhibit neuroinflammation and oxidative stress in Alzheimer’s disease [74,75].

### 3.4. Limitations

Several limitations of this study should be acknowledged. First, while APP/PS1 transgenic mice are widely used AD models, they primarily recapitulate Aβ pathology and do not fully replicate the tau pathology and neuronal loss seen in human AD. Second, the in vivo experiments were conducted in young adult mice (6 months old), whereas AD primarily affects elderly populations; aged mice might exhibit different responses. Third, our study focused on short-term treatment (4 weeks), and long-term safety and efficacy remain to be evaluated. Long-term toxicity, biodistribution, biodegradation, and pharmacokinetic analyses are essential to fully assess the translational safety of MXene-based nanomaterials for clinical AD therapy. Fourth, the mechanism by which Ti2C@BSA-NBP crosses the blood–brain barrier was not directly investigated in this study. While in vivo fluorescence imaging demonstrated brain accumulation of Ti2C@BSA, these experiments utilized precursor materials rather than the final Ti2C@BSA-NBP composite, and quantitative biodistribution analysis was not performed. Additionally, in vitro BBB model experiments (e.g., bEnd.3 endothelial cell Transwell system) and receptor-mediated transport inhibition studies were not conducted. BSA has been reported to facilitate BBB crossing via gp60 receptor-mediated transcytosis and SPARC-mediated albumin uptake [76,77], which may partially explain the observed brain enrichment; however, the exact transport mechanism of Ti2C@BSA-NBP warrants further investigation. Future studies will include quantitative biodistribution analysis of Ti2C@BSA-NBP in major organs, in vitro BBB permeability assays using Transwell models, and competitive inhibition experiments to elucidate the BBB penetration pathway. Fifth, clinical translation would require evaluation in larger animal models and consideration of immunogenicity concerns with bovine-derived BSA. Sixth, while FTIR spectroscopy provided evidence for amide bond formation between BSA-NBP and Ti2C, X-ray photoelectron spectroscopy (XPS) characterization, which could offer more definitive elemental-level evidence of covalent bonding, was not performed in this study. Future studies will include XPS analysis to further confirm the chemical linkage between BSA-NBP and Ti2C. Seventh, stability assessment was limited to PBS buffer conditions, and the colloidal stability of Ti2C@BSA-NBP in biological media containing serum proteins was not evaluated. Protein corona formation in serum may alter the hydrodynamic size, surface charge, and drug release kinetics of the nanocomposite. Future studies will include stability testing in fetal bovine serum (FBS)-containing media and simulated physiological fluids to better predict in vivo behavior. 

### 3.5. Innovation and Clinical Significance

Despite these limitations, this study makes several important contributions. The innovation of Ti2C@BSA-NBP lies in the synergistic combination of three therapeutic elements: (1) MXene providing multi-enzyme antioxidant activity and potential BBB transport capability; (2) BSA serving as a biocompatible carrier that improves drug loading and reduces immunogenicity; and (3) NBP offering neuroprotective effects against Aβ aggregation and neuroinflammation. The establishment of Ti2C@BSA-NBP represents a new approach to address the poor delivery and bioavailability of AD drugs in addition to the mitigation of major pathological characteristics of AD, such as oxidative stress, ferroptosis, and neuronal injury. This study is particularly oriented towards clinical application by providing a new, efficient, and safe method for the treatment of AD, a neurological disorder lacking effective disease-modifying treatments. In the future, more attention should be paid to the optimization of drug loading and release behaviors of Ti2C@BSA-NBP, while comprehensive studies on its safety and efficacy in larger animal models as well as clinical trials are warranted. Moreover, the application of MXenes in neural tissue engineering extends beyond AD therapy. Recent studies have demonstrated that MXene-based materials can promote neural stem cell proliferation and differentiation, enhance electrophysiological maturation of neurons, and facilitate neural tissue regeneration in various neurological disorder models [78]. The intrinsic enzyme-mimicking and ROS-scavenging properties of MXenes make them particularly suitable for neuroprotective applications, as oxidative stress is a common pathological feature across neurodegenerative diseases. These broader applications in neural repair further support the therapeutic potential of our Ti2C@BSA-NBP composite for AD treatment.

To further clarify the mechanism underlying the anti-ferroptotic effects of Ti2C@BSA-NBP, we performed additional mechanistic validation experiments (Figure 7). The results showed that H2O2 exposure markedly suppressed nuclear Nrf2 (0.388 ± 0.070 of Control) and downstream HO-1, SLC7A11, and GPX4 expression, while strongly inducing lipid peroxidation (4.658 ± 1.851 of Control). Ti2C@BSA-NBP treatment significantly reversed these changes, restoring Nrf2/HO-1/SLC7A11/GPX4 expression to 0.843 ± 0.153/0.855 ± 0.155/0.800 ± 0.145/0.838 ± 0.152 of Control and reducing lipid ROS to 1.952 ± 0.776 of Control. Importantly, the Ferrostatin-1 positive control also reduced lipid ROS (1.494 ± 0.594 of Control), supporting that the protective effect of Ti2C@BSA-NBP is mediated, at least in part, through ferroptosis inhibition. However, Fer-1 did not restore Nrf2/HO-1/SLC7A11 expression, whereas Ti2C@BSA-NBP did, indicating that Ti2C@BSA-NBP provides broader protection through both direct ferroptosis suppression and Nrf2 pathway activation.

## 4. Materials and Methods

### 4.1. Material Preparation

The first step in the preparation of 2D MXene-loaded butylphthalide (NBP) nanomaterial was the activation of 60 mg of bovine serum albumin (BSA; Sigma, Sigma, Kanagawa, Japan; CAS 9048-46-8), which was dissolved in 1.5 mL ultrapure water together with 30 mg of sodium dodecyl sulfate (SDS; Aladdin Chemistry Co., Ltd., Shanghai, China; CAS 151-21-3) and 2.2 mg of dithiothreitol (DTT; Aladdin Chemistry Co., Ltd., CAS 3483-12-3) in a sample bottle. This mixture was stirred in an oil bath at 200 rpm at 90 °C for 2 h, followed by storage at 4 °C. MES buffer (VETEC/Sigma, CAS 4432-31-9) was prepared at a final concentration of 0.47 g/mL in 50 mL ultrapure water and adjusted to pH 4.25–4.8 with 0.1 M sodium hydroxide (Aladdin Chemistry Co., Ltd., CAS 1310-73-2). A 4 mg/mL NBP solution was prepared in ultrapure water. For BSA-NBP nanoparticle formation, 25 µL of activated BSA solution was mixed with 950 µL MES buffer and 25 µL NBP solution (4 mg/mL), and the mixture was incubated at 37 °C with shaking at 800 rpm for 4 h. The product was purified by ultrafiltration using a 100 kDa molecular weight cut-off (MWCO) centrifugal filter at 4000 r/min (approximately 2000× *g*; rotor radius: 11.5 cm) for 10 min and stored at 4 °C. For the synthesis of Ti2C@BSA-NBP, 1 mg/mL Ti2C-COOH (North Cornell, Ithaca, NY, USA) was first activated with EDC (Aladdin) at a molar ratio of EDC:Ti2C-COOH = 10:1 for 1 h at room temperature, followed by addition of NHS (Aladdin Chemistry Co., Ltd., CAS 6066-82-6) at a molar ratio of NHS:EDC = 1:1 for 1 h. The activated Ti2C was then mixed with BSA-NBP solution at a mass ratio of Ti2C:BSA-NBP = 1:1 and incubated at 4 °C for 12 h. The final product was purified by ultrafiltration (100 kDa MWCO, 4000 r/min, 10 min) and stored at 4 °C.

### 4.2. Characterization of 2D MXene-Loaded Butylphthalide (NBP) Nanomaterials

Two-dimensional MXene-loaded butylphthalide (NBP) nanomaterials were characterized through systematic methods to characterize the encapsulation efficiency, particle size, and stability. The encapsulation efficiency and loading rate were evaluated using the differential method. As a specific case, 5 mg NBS standard substance was measured into a beaker and a volumetric flask, and adequate ultrapure water was added for complete dissolution. The volume of this solution was then adjusted to a 5 mL volumetric flask, where the solution was made of 1 mg/mL NBP. Serial dilutions were performed to prepare different concentrations, including 15.625, 31.25, 62.5, 125, 250, and 500 µg/mL. A UV-2600i ultraviolet spectrophotometer (Shimadzu, Kyoto, Japan) was used to measure the absorbance at 275 nm, and by plotting concentration on the horizontal axis and absorbance on the vertical axis, a standard curve was prepared.

### 4.3. Encapsulation Efficiency (EE) and Loading Efficiency (LE)

Then, we collected solutions above and below the ultrafiltration tube and measured their volumes. Appropriate dilution of the solution below ultrafiltration was performed, and its absorbance was measured using UV-2600i. The mass of the drug in the filtrate was calculated from equations of the standard curve prepared before by substituting the values into it. The difference method was used to find the amount of loaded drug. The encapsulation efficiency (EE) and loading efficiency (LE) were computed through the formulas: the amounts known as the ratio of loaded drug mass to total drug mass EE and the ratio of the mass of loaded drug to the sum of the mass of loaded drug and mass of carrier LE are used.

### 4.4. Measure Their Zeta Potential and Their Particle Size

Besides encapsulation assessment, the particle size and Zeta potential of the samples were characterized using the Dynamic light scattering (Zeta potential) Malvern Nano ZS90 (Malvern Instruments, Malvern, UK). Deionized water was added to a concentration to dilute the samples, and they were uniformly dispersed. After that, we placed them in their specific sample cells in order to measure their Zeta potential and their particle size through the two modes available on the Malvern device. All measurements were performed at 25 °C with a detection angle of 90°. Each sample was measured in triplicate (*n* = 3), and results are expressed as mean ± standard deviation (SD). The polydispersity index (PDI) was also recorded to evaluate the uniformity of particle size distribution.

### 4.5. Fourier-Transform Infrared Spectroscopy (FTIR) Analysis

In addition, Fourier-transform infrared spectroscopy (FTIR) was used to probe the molecular structure of the nanomaterials. For this analysis, this sample and potassium bromide (KBr) were dried in a vacuum oven at 80 °C for 12 h. The powders were then ground thoroughly in an agate mortar and pressed into tablets for examination by a Thermo Scientific Nicolet iS20 infrared spectrometer (Thermo Fisher Scientific, Waltham, MA, USA).

### 4.6. X-Ray Diffraction (XRD) Analysis

The MXene-loaded NBP nanomaterials were analyzed via X-ray diffraction (XRD) to analyze the crystallographic structure. Freeze, dried, and made into fine powder. phase identification was by X-ray diffraction employing a Rigaku Ultima IV X-ray diffractometer (Rigaku Corporation, Tokyo, Japan) on the resulting powder.

### 4.7. Transmission Electron Microscopy (TEM) Analysis

Transmission electron microscopy (TEM) visualized the morphology of the nanomaterials. An amount of 70 µL of 2% uranyl acetate for negative staining was added, and another 3 min later, the excess was removed. Afterward, the samples were naturally dried. Finally, a stability test was performed to evaluate the stability of the MXene@BSA-NBP nanodrug delivery system. BSA@MXene nanoparticles and phosphate-buffered saline (PBS) solution were mixed to a specific amount and allowed to stand at room temperature. The stability of our sample was measured over time by measuring the particle size at 0, 12, 24, and 48 h. The release behavior of the MXene@BSA NBP nanodrug system was evaluated in an in vitro drug release experiment. The drug delivery system was sealed in a 2 mL amount and placed in a sample bottle with 10 mL of pH 7.4 buffer and continuously shaken at 37 °C and 150 rpm. In between, every two hours, and after ten hours every five hours, 2 mL of solution was centrifuged. The concentration in the HPLC supernatant was subsequently used to calculate the cumulative release amount of the drug at different time points.

### 4.8. Cell Culture

Cell culture induced by H2O2 was examined in a PC12 cell line purchased from the Cell Bank of the Chinese Academy of Sciences (CBTCCCAS), and the effect of different nanoparticle treatments was examined. DMEM supplemented with 10% FBS, 50 U/mL penicillin, and 50 μg/mL streptomycin was used to culture rat cells, which were maintained in a cell culture incubator at 37 °C and 5% CO2 and subcultured weekly to maintain the cells in an early passage state. At approx. 80% confluence, cells in the medium were trypsinized (0.05%, *w*/*v*) and transferred to new 35 mm-diameter dishes every 2 days. The cellular H2O2-injury model was constructed by treating the cells with 200 μM H2O2 (Sinopharm Chemical Reagent Co., Ltd., Beijing, China) for 4 h for subsequent experiments. The cells were divided into six experimental groups: (1) Control, untreated cells; (2) H2O2, cells treated with 200 μM hydrogen peroxide; (3) NBP, cells treated with pure butylphthalide; (4) Ti2C, cells treated with Ti2C nanoparticles; (5) BSA-NBP, cells treated with BSA-conjugated NBP nanoparticles; and (6) Ti2C@BSA-NBP, cells treated with Ti2C nanoparticles loaded with BSA-conjugated NBP. All these groups had at least three independent replicates in the study. PC12 cells, a rat pheochromocytoma cell line, were chosen as the in vitro model because they exhibit neuronal characteristics and have been widely used to study neurotoxicity and neuroprotection in the context of AD and oxidative stress. The H_2_O_2_ concentration (200 µM) and exposure time (24 h) were selected based on preliminary dose–response experiments to induce significant oxidative damage without excessive cell death.

### 4.9. Cell Counting Kit-8 Assay

Firstly, cells were cultured using a 96-well plate, with 200 µL of a cell suspension at a density of 5000 cells per well and then allowed to adhere at 37 °C, 5% CO2 in an incubator with saturated humidity. Afterward, the media were removed from cells and treated with different materials for different times (0, 12 h, 24 h, 48 h), with control treatment wells containing just media. After the treatment period, the medium was discarded, and 20 µL CCK-8 (cell counting kit-8, Sigma-Aldrich) was added to each well at 37° C for a further 2 h to produce formazan dye. Absorbance at 450 nm was subsequently measured using a microplate reader. Absorbance values were recorded, and cell viability was calculated as a percentage of viability.

### 4.10. Phagocytosis Assay

The phagocytosis of materials by cells was observed by fluorescence microscopy and detected by flow cytometry. The cells were seeded at a concentration of 3.5 × 10^5^ cells per well in 6-well plates and maintained at 37 °C, 5% CO2, and saturated humidity. Fluorescein isothiocyanate (FITC)-labeled materials were added, and cells were allowed to incubate with those for 0, 4, and 8 h periods until cells reached 70% confluence. FITC-labeled NPs in cells were quantified by flow cytometry, and DAPI (1:1000) was added to cells for staining for 15 min. After washing, CLSM was used to monitor the uptake of nanoparticles by cells.

### 4.11. Reactive Oxygen Species Staining

Exponentially growing cells were planted into a 48-well plate at a density of 5 × 10^4^ cells per well and left to grow for 12 h at 37 °C, 5% CO2, and saturated humidity. Then, different materials in each group were added and incubated with the cells for 24 h. After this exposure, 4 h oxidative stress was then induced by the addition of H_2_O_2_ to a final concentration of 200 μM. Next, the cells were incubated with a 20 μM solution of DCFH-DA for 30 min at 37 °C, and green fluorescence was imaged by fluorescence microscope. The fluorescence intensity was measured by Image J. Untreated cells were used as controls. The results are expressed as the mean fluorescence intensity (MFI) of dichlorofluorescein (DCF).

### 4.12. JC-1 Staining

Cells were cultured under various conditions, such as exposure to different materials, as well as 200 µM H_2_O_2_, to assess the mitochondrial membrane potential (MMP). Next, a volume of 1 mL of JC-1 staining solution was added and then mixed gently; then, the specimen plate was incubated with a cell culture incubator (37 °C, 20 min). During incubation, a JC-1 staining buffer (1X) was made from four parts distilled water and one part JC-1 staining buffer (5X), and it was placed in an ice bucket. The cell pellet was first washed twice with JC-1 staining buffer (1X), incubated, and washed to withdraw supernatant containing purified protein. Finally, 2 mL of cell culture medium with or without serum and phenol red was added, and the cells were examined by fluorescence or confocal laser scanning microscopy.

### 4.13. Iron Ion Level Detection

FerroOrange (Dojindo Laboratory, Kumamoto, Japan), following the manufacturer’s protocols, was used to assess the intracellular iron concentration. Dishes containing seeded cells were stained with 1 µM FerroOrange for 30 min at 37°C. Next, cells were washed with PBS and analyzed on a confocal microscope. The Colorimetric Iron Assay Kit (Ab83366, Abcam) was used to quantify the Fe^2+^ levels in brain tissue. The OD values at 593 nm were measured, and the levels of relative were determined. Concentrations were quantified in terms of µmol/kg wet weight for each optical density OD.

### 4.14. In Vitro Immunofluorescence Staining

After 15 min of fixation using 4% paraformaldehyde Sigma at room temperature and 10 min of permeabilization with 0.1% TritonX-100 Thermo Fisher Scientific at room temperature, cells were rehydrated with the buffer used above and washed with 1X PBS. The cells were preincubated for one hour with 5% bovine serum albumin (BSA) (Sigma-Aldrich) to block nonspecific binding. After overnight exposure at 4 °C to primary antibodies, including the primary anti-GPX4 antibody (1:100, Ab83366, Abcam) and ACSL4 Polyclonal Antibody (1:200, PA5-27137, Invitrogen, Carlsbad, CA, USA), the cells were washed with phosphate-buffered saline (PBS) and probed with Goat Anti–Mouse IgG H&L (Alexa Fluor^®^ 488) (ab150115, Abcam) for 1 h overnight in the dark. Next, the nuclei were stained with 4’,6-diamidino-2-phenylindole (DAPI) solution (Shanghai Biyuntian Biotechnology Corporation, Shanghai, China; #P0131). Quantification and analysis of GPX4 and ACSL4 expression were performed using Olympus CX43 Microscope (Olympus Corporation, Tokyo, Japan).

### 4.15. Animals

As a mouse model of Alzheimer’s disease (AD) amyloid plaques, we used 6-month-old APP/PS1 transgenic mice (*n* = 30). APP/PS1 double transgenic mice express a chimeric mouse/human amyloid precursor protein (Mo/HuAPP695swe) and a mutant human presenilin 1 (PS1-ΔE9), both directed to CNS neurons. These mice develop age-dependent Aβ plaque deposition beginning at approximately 4–6 months of age, with robust amyloid pathology by 6 months, making them suitable for evaluating therapeutic interventions targeting Aβ accumulation and related pathological processes. C57BL/6 mice (*n* = 6) of the same age were used as wild-type controls. The mice (male, weight 18 ± 2 g) were purchased from Beijing Weitonglihua Experimental Animal Technology Co., Ltd., (Beijing, China) with the experimental animal production license number SCXK(Shanghai)2024-0001. The mice were maintained in a standard animal room, with a normal temperature (20 °C–25 °C), relative humidity of about 30–70%, and a light/dark cycle of 12 h/12 h. Animals were provided with unlimited access to food and water. We attempted to use the lowest possible numbers of mice and cause the least amount of suffering for our animals. All animal procedures were in accordance with animal ethics. After 7 days of adaptive feeding, it was found that the Control group was in a good mental state, ate normally, and was active, while the AD group was in a depressed mental state, ate less, was inactive, and lay still. There was no death in each group. The mice were divided into 6: Control, AD, NPB (nanoparticles), Ti_2_C, BSA–NBP, and Ti_2_C@BSA–NBP. The mice in the AD group were fed for 2 weeks and then grouped for treatment. The mice in the different material treatment groups were injected with 200 μL/mouse through the tail vein, and the AD group was injected with the same dose of normal saline twice a week. After 4 weeks of continuous treatment, brain tissue and blood were obtained for subsequent experiments. The brain tissue was stored in paraformaldehyde or liquid nitrogen, the blood was immediately separated, and the serum was stored at −80°C for later use.

### 4.16. In Vivo Imaging

Via tail vein, 500 nM of Cy5 or Cy5-labeled Ti2C and Ti2C@BSA nanoparticles was administered to mice. Fluorescence was detected using a multi-mode in vivo imaging system (Model: B Pro, Wuhan BIOVIVO, Wuhan, China). Briefly, mice were shaved and then imaged with a 200 mm field of view. The brain was excited with an excitation wavelength of 630 nm and a bandwidth of 30 nm for emission to be detected at a primary wavelength of 680 nm with a bandwidth of 40 nm. An exposure time of 1 s was employed for acquisition of the fluorescence signal.

### 4.17. Barnes Maze Test

Mice in each of the groups were assessed using the Barnes maze test to evaluate spatial memory. The maze was constructed from 1 m diameter circular white acrylic slab, 6 mm-thick. Holes of diameter 25 cm were placed 2.5 cm from the edge. A stool with a circular platform 60 cm above the ground was mounted. The plastic dark box escape cage was changed after the test of each animal. The maze was located in a dedicated room and bathed, on the top, by two 120 W lamps about 2 m above the floor, with entry through a side oriented upwards. Visual cues such as colored paper shapes (squares, triangles, circles) were projected around the room. The mice were tested in the maze one at a time, and the maze was cleaned with 70% ethanol after every test, as well as rotated 3 holes clockwise to hone in on the smell cues. All sessions were videotaped and analyzed with smart V2.5.21 software (Panlab, Barcelona, Spain). The animals went through three phases: habituation, acquisition training, and acquisition probe. Each experimental session ended by placing the mouse in a holding cage and cohabitating all mice in a home cage at the end of the day. In the habituation phase of the first day, the mouse entered the escape tunnel for 1 min and then performed a free exploration of the maze for a maximum of 5 min or until entering the escape tunnel. We turned off the buzzer during habituation but turned on the bright light. The mouse was not permitted to enter the tunnel unless coerced, at which point it was nudged into the cage and left for 1 min. The first acquisition training session was 1.5 h after habituation. The mouse was put on top of a container-covered platform in the center of the maze. After 10 s of inhibitory tone, the buzzer was turned on, but the mouse was allowed to explore for either 3 min or until it entered the escape tunnel. If the mouse was not in the escape tunnel after 3 min, the mouse was placed inside the escape tunnel for 30 s. Two 1.5 h acquisition training sessions were performed twice per day on days 2 and 3. There were three acquisition training sessions at 15 min intervals under the same conditions on day 4. On day 5 was the acquisition probe session. The escape cage was taken away, and after 10 s, the buzzer was turned on, and the mouse was left to explore the maze for 90 s before being placed back in its holding cage.

### 4.18. Elevated Plus Maze

An Elevated Plus Maze analysis instrument (EPM) was used to quantify the anxiety of mice. The EPM had 2 open arms (arm length 50 × arm width 10 cm) and 2 closed arms (arm length 50 × arm width 10 × arm height 29 cm) and a central area (10 × 10 cm). Once the animal was placed in the center facing towards the open arm and we verified that the camera system was in place, we recorded the behavior change of the animals over the next 5 min. The time to enter the open and closed arms was measured from the recorded footage. After completing the animal experiment, feces from the maze were quickly removed, and the bottom of the tank was washed down with 75% ethanol. Additionally, the tank was gently wiped with clean gauze so that any remaining olfactory traces from animals would be eliminated to prevent them from interfering with later experiments. This environment had to be quiet throughout the experiment to avoid external interference in the behavior of mice and to obtain correct results without unnecessary sounds and disturbances around the experimental setup.

### 4.19. Hematoxylin and Eosin Staining

Healthy mouse tissues, including the hippocampal brain and major organs (heart, liver, spleen, lungs, and kidneys), were collected and fixed in 10% neutral buffered formalin (Thermo Fisher Scientific, Waltham, MA, USA) for 24 h. The samples were then fixed and embedded in paraffin wax, sectioned to a thickness of 5 μm. Afterward, sections were deparaffinized with xylene (Sinopharm Group Co., Ltd., Shanghai, China) and rehydrated in a series of graded alcohols (100, 95, 70, and 50% ethanol) and distilled water. The sections were immersed in 0.5% hematoxylin (Sinopharm Group Co., Ltd., Shanghai, China) for 5 min, differentiated in 1% hydrochloric acid in 70% ethanol, and counterstained in 1% eosin (Sinopharm Group Co., Ltd., Shanghai, China) for 2 min. The sections were washed in distilled water, dehydrated in graded alcohol, cleared with xylene, and finally mounted with a mounting medium (Permount, Fisher Scientific, Pittsburgh, PA, USA). Any histopathological changes or signs of toxicity were assessed by examination of the stained slides with a standard light microscope (Olympus, Tokyo, Japan).

### 4.20. Nissl Staining

The brain was removed, fixed with 4% paraformaldehyde overnight, dehydrated in 30% sucrose in 4% paraformaldehyde, and then sliced into 5 μm slices with a vibratome (VT 1200S, Leica, Wetzlar, Germany). The sections were coated with gelatin and placed into chloroform alcohol (1:1) overnight. The following day, the sections were dehydrated in the graded ethanols (that is, 100%, 95%). The slices were scratched by a platinum wire knife to approximately 1.0 mm-thick sections and incubated in 0.1% cresyl violet acetate for 10 min, washed quickly with distilled water, and differentiated in 95% ethyl alcohol for 30 min. They were then dehydrated in graded ethanols and coverslipped with PermountTM. The slices were visualized and scanned by a slide scanner (Slide Scan System SQS1000, Teksqray, Guangzhou, China). Quantitative analysis of Nissl staining was performed using ImageJ software Version 1.54 (NIH) to measure the optical density of Nissl-positive staining in the hippocampal DG, CA1, and CA3 regions. All quantitative analyses were performed by a blinded observer.

### 4.21. Transmission Electron Microscopy

Tissues were quickly extracted into the brain and fixed in a solution of 2.5% glutaraldehyde (EM Sciences, Hatfield, PA, USA) and 4% paraformaldehyde (Electron Microscopy Sciences, Hatfield, PA, USA) in 0.1 M phosphate buffer (pH 7.4) for 2 h at 4 °C. Following fixation, the tissues were washed three times for 10 min each in 0.1 M phosphate buffer and then post-fixed in 1% osmium tetroxide (OsO4; Electron Microscopy Sciences) for 1 h at room temperature. After another series of washes, samples were dehydrated through graded ethanol solutions (30%, 50%, 70%, and 100% for 10 mn each) and embedded in Epon resin EMBED 812 kit, EMS). To facilitate further imaging studies, thin sections (70 nm) were cut using an ultramicrotome (Leica Ultracut UCT) and collected on copper grids. To visualize mitochondrial structures, grids were stained with uranyl acetate (15 min; Sigma-Aldrich, St. Louis, MO, USA) and lead citrate (10 min; Sigma-Aldrich) and examined by Transmission Electron Microscopy (TEM; JEOL JEM-1400) at 120 kV. Quantitative morphometric analysis of mitochondria was performed using ImageJ software (NIH) on TEM micrographs. Parameters measured included mitochondrial area, aspect ratio (major/minor axis), circularity (4 × pi × area/perimeter^2), and cristae density. Mitochondria were classified as elongated (aspect ratio ≥ 2.0), intermediate (1.5 ≤ AR < 2.0) or swollen (AR < 1.5). All analyses were performed by a blinded observer.

### 4.22. Enzyme-Linked Immunosorbent Assay (ELISA)

Mouse brain tissue homogenate samples and cell culture supernatants were used to quantify the expressed levels of IL-1β, IL-6, and TNF-α using mouse enzyme-linked immunosorbent assay (ELISA) kits (Mouse IL-1β ELISA Kit (CME0015, 4abio/Four-star cypress), Mouse IL-6 ELISA kit (CME0006, 4abio/Four-star cypress), and Mouse TNF-a ELISA Kit (CME0004, 4abio/Four-star cypress)) according to the manufacturer’s protocols. Each cytokine antibody was attached to its corresponding ELISA plate (Thermo Fisher Scientific), the samples and standards were added and incubated at room temperature for 2 h. Subsequently, we washed and then added detection antibodies and Goat anti Mouse IgG H&L (HRP) (ab205719, Abcam). Absorbance was measured at 450 nm with a microplate reader (RT6100; Rayto, Shenzhen, China) after the enzymatic reaction, and the samples and standards were added and incubated at room temperature for 2 h. Next, after washing, we added detection antibodies and Goat Anti-Mouse IgG H&L (HRP) (ab205719, Abcam). After the enzymatic reaction, absorbance was measured at 450 nm using a microplate reader (RT6100; Rayto, Shenzhen, China). The concentrations of cytokines in mouse brain samples were determined by analyzing the data using appropriate software.

### 4.23. Detection of GSH–PX and MDA

For evaluating oxidative stress, GSH–PX and MDA were detected in brain tissue homogenates and cell culture supernatant. The levels of GSH–PX were quantified using a GSH–PX assay kit (#A005-1-2, Nanjing Jiancheng, Nanjing, China) according to the manufacturer’s protocols. Additionally, MDA levels were determined by an MDA assay kit (#A003-1-2, Nanjing Jiancheng, Nanjing, China). Absorbance values were detected using a microplate reader (RT6100; Rayto) at 450 nm, and the assays were repeated in triplicate.

### 4.24. Immunofluorescence Staining of Brain Tissues

Brain tissues were fixed in 4% paraformaldehyde, paraffin-embedded, and cut into 3 µm-thick slices. After deparaffinized slices were exposed to primary antibodies for 30 min at room temperature after being blocked with 3% BSA, namely anti-GPX4 (1:500, sc-166120, Santa Cruz Biotechnology, Dallas, TX, USA), ACSL4 (1:500, sc-365230, Santa Cruz Biotechnology), and anti- Aβ (β-Amyloid) (1:500, sc-374527, Santa Cruz Biotechnology) antibodies. Treated with a secondary antibody for 1 h at room temperature the saturated slides. A DAPI fluorescent stain was used to stain the nuclei. The cells were observed under an LSM800 confocal microscope from Carl Zeiss (Oberkochen, Germany).

### 4.25. Western Blotting

We collected samples we had of hippocampal brain tissues or cells on ice and suspended the tissues or cells in 50 uL of RIPA lysis buffer solution (50 mM Tris-HCl, 100 mM NaCl, 1% Triton X-100, 5 mM EDTA, 1 mM PMSF). Separation of the mixture was performed by centrifuging it at a speed of 12,000 g for 15 min, and the supernatant was subjected to soluble protein analysis. The BCA assay method was used to assess the protein concentration. Proteins from each sample were then loaded on 8–15% SDS-PAGE gels and electrophoresed and transferred to 0.45 μm polyvinylidene fluoride membranes (Merck Millipore, Burlington, MA, USA) in equal amounts. In an attempt to minimize non-specific binding, membranes were treated with a 5% nonfat milk solution and incubated with the primary and then horseradish peroxidase-linked secondary antibodies (goat anti-mouse IgG-HRP (1:5000, Thermo Fisher Scientific, 31430)). Quantification of signal intensities of immunoblots was quantified using Image J software (National Institutes of Health, NIH, Bethesda, MD, USA) and visualized with ECL (ECL kit, Pierce Thomson Biotechnology (Beijing) Co., Ltd., Hangzhou, China) using a luminometer (ChemiScope 6000, Clinx, Shanghai, China). Primary antibodies included rabbit anti-GPX4 (1:1000, Abcam, ab125066), rabbit anti-ACSL4 (1:1000, Sigma-Aldrich, HPA016270), and mouse anti-Aβ (1:1000, Millipore, MABN262). Image quantification was performed using ImageJ software.

### 4.26. C11-BODIPY 581/591 Staining for Lipid ROS Detection

Lipid peroxidation was detected using the C11-BODIPY 581/591 fluorescent probe (Invitrogen, D3861). PC12 cells were seeded in 24-well plates containing glass coverslips and treated with the indicated materials for 24 h, followed by exposure to 200 μM H2O2 for 4 h. Cells were then incubated with 2 μM C11-BODIPY 581/591 in serum-free medium at 37 °C for 30 min in the dark, washed three times with PBS, fixed with 4% paraformaldehyde for 15 min, and counterstained with DAPI. Fluorescence images were captured using a fluorescence microscope (excitation/emission: 488/510 nm for oxidized green and 581/591 nm for reduced red). The green/red fluorescence intensity ratio was quantified using ImageJ software (*n* = 5 randomly selected fields per group). All experiments were performed with six independent biological replicates (*n* = 6).

### 4.27. Western Blot Analysis of the Nrf2/HO-1/SLC7A11/GPX4 Axis

Nuclear and cytoplasmic proteins were extracted from PC12 cells using a Nuclear and Cytoplasmic Protein Extraction Kit (Beyotime, Shanghai, China; P0027) according to the manufacturer’s instructions. Protein concentrations were determined by the BCA assay. Equal amounts of protein (30 μg per lane) were separated by 10% SDS-PAGE and transferred to PVDF membranes. Membranes were blocked with 5% nonfat milk for 1 h and incubated overnight at 4 °C with primary antibodies against Nrf2 (1:1000, Abcam, ab62352), HO-1 (1:1000, Abcam, ab68477), SLC7A11 (1:1000, Abcam, ab175186), and GPX4 (1:1000, Abcam, ab125066). Histone H3 (1:1000, CST, #4499) and β-actin (1:1000, CST, #4970) were used as loading controls for nuclear and cytoplasmic fractions, respectively. After incubation with HRP-conjugated secondary antibodies, protein bands were visualized using an ECL detection kit and quantified by ImageJ. All experiments were performed with six independent biological replicates (*n* = 6).

### 4.28. Statistics

GraphPad Prism 8.0 statistical software was used for data analysis. Data were expressed as mean ± SD. The normality of all data was assessed using the Shapiro–Wilk test (for sample sizes *n* ≤ 50) or the Kolmogorov–Smirnov test (for sample sizes *n* > 50). For data that passed the normality test, the differences between groups were compared using one-way ANOVA followed by Tukey’s post hoc test for pairwise comparisons, or two-way ANOVA followed by Bonferroni’s post hoc test when two factors were involved. For data that did not follow a normal distribution, the Kruskal–Wallis test followed by Dunn’s post hoc test was used. The difference was considered statistically significant at *p* < 0.05.

## 5. Conclusions

Overall, this work reports a new composite material (Ti2C@BSA-NBP), which shows good antioxidant, anti-ferroptosis, and neuroprotection effects. This composite biomaterial proves to be a useful potential new therapy in the treatment of Alzheimer’s disease due to its potential to deliver the drug specifically to the affected part of the brain.

## Data Availability

The original contributions presented in this study are included in the article/Supplementary Material. Further inquiries can be directed to the corresponding author

## Supplementary Materials

The following supporting information can be downloaded at: https://www.mdpi.com/article/10.3390/ph19091386/s1. Figure S1. XRD patterns of Ti2C and Ti2C@BSA-NBP. The XRD analysis demonstrates that the crystalline structure of Ti2C was largely retained after grafting with BSA-NBP, indicating that the amidation reaction did not substantially alter the crystallographic structure of Ti2C. Figure S2. Encapsulation efficiency and loading rate of BSA-NBP nanoparticles. The encapsulation efficiency (EE) and loading efficiency (LE) were determined using the ultrafiltration-differential method. The loading rate was 12.98% and the encapsulation rate was 37.28%. Figure S3. In vivo fluorescence imaging of Cy5-labeled Ti2C and Ti2C@BSA in mice. (A) Whole-body fluorescence imaging at 4 h and 12 h after tail vein injection. (B) Ex vivo organ imaging showing the biodistribution in major organs including brain, heart, liver, spleen, lung, and kidney. Both Ti2C and Ti2C@BSA showed brain accumulation, with Ti2C@BSA exhibiting relatively higher brain enrichment. Figure S4. H&E staining of major organs from mice in each group. Representative histological sections of heart, liver, spleen, lung, and kidney showed no obvious pathological changes, indicating that the materials in each group were non-toxic and exhibited good biosafety. Scale bars are indicated on the images. Figure S5. Quantitative analysis of Nissl staining and mitochondrial morphology. (A) Quantitative analysis of Nissl staining optical density in the DG, CA1, and CA3 regions of the hippocampus. (B) Quantitative morphometric analysis of hippocampal mitochondria, including mitochondrial area, aspect ratio, circularity, and cristae density. Data are expressed as mean ± SD (n = 3–6). * *p* < 0.05, ** *p* < 0.01.

## Abbreviations

The following abbreviations are used in this manuscript: AD, Alzheimer’s Disease; ROS, Reactive Oxygen Species; NBP, Butylphthalide; BSA, Bovine Serum Albumin; Aβ, Amyloid-beta; BBB, Blood–brain barrier; SDS, Sodium Dodecyl Sulfate; DTT, Dithiothreitol; MES, 2-(N-morpholino)ethanesulfonic acid; PBS, Phosphate-Buffered Saline; TEM, Transmission Electron Microscopy; FTIR, Fourier-transform infrared spectroscopy; XRD, X-ray diffraction; CCK-8, Cell Counting Kit-8; FITC, Fluorescein isothiocyanate; DAPI, 4’,6-diamidino-2-phenylindole; CLSM, Confocal Laser Scanning Microscopy; DCFH-DA, Dichlorofluorescein diacetate; DCF, Dichlorofluorescein; SOD, Superoxide Dismutase; CAT, Catalase; GPx, Glutathione Peroxidase; GSH, Glutathione; GSSG, Oxidized Glutathione; GR, Glutathione Reductase; NADP, Nicotinamide adenine dinucleotide phosphate; MDA, Malondialdehyde; ELISA, Enzyme-Linked Immunosorbent Assay; TNF-α, Tumor Necrosis Factor alpha; IL-1β, Interleukin-1 beta; IL-6, Interleukin-6; H&E, Hematoxylin and Eosin; EPM, Elevated Plus Maze; GPX4, Glutathione Peroxidase 4; ACSL4, Acyl-CoA Synthetase Long Chain Family Member 4; C11-BODIPY, C11-BODIPY 581/591 (lipid peroxidation-sensitive fluorescent probe).

## References

[1] Lage J.M. (2006). 100 Years of Alzheimer’s disease (1906–2006). J. Alzheimer’s Dis..

[2] Sun B.L., Li W.W., Zhu C., Jin W.S., Zeng F., Liu Y.H., Bu X.L., Zhu J., Yao X.Q., Wang Y.J. (2018). Clinical Research on Alzheimer’s Disease: Progress and Perspectives. Neurosci. Bull..

[3] Tiwari S., Atluri V., Kaushik A., Yndart A., Nair M. (2019). Alzheimer’s disease: Pathogenesis, diagnostics, and therapeutics. Int. J. Nanomed..

[4] Rajmohan R., Reddy P.H. (2017). Amyloid-Beta and Phosphorylated Tau Accumulations Cause Abnormalities at Synapses of Alzheimer’s disease Neurons. J. Alzheimer’s Dis..

[5] Lee T.S., Krishnan K.R. (2010). Alzheimer’s disease-the inexorable epidemic. Ann. Acad. Med. Singap..

[6] Jaroudi W., Garami J., Garrido S., Hornberger M., Keri S., Moustafa A.A. (2017). Factors underlying cognitive decline in old age and Alzheimer’s disease: The role of the hippocampus. Rev. Neurosci..

[7] Bhatt J., Comas Herrera A., Amico F., Farina N., Wong J., Orange J., Gaber S., Knapp M., Salcher-Konrad M., Stevens M. (2019). The World Alzheimer Report 2019: Attitudes to Dementia.

[8] Bonda D.J., Lee H.G., Blair J.A., Zhu X., Perry G., Smith M.A. (2011). Role of metal dyshomeostasis in Alzheimer’s disease. Metallomics.

[9] Babić Leko M., Langer Horvat L., Španić Popovački E., Zubčić K., Hof P.R., Šimić G. (2023). Metals in Alzheimer’s Disease. Biomedicines.

[10] Islam F., Shohag S., Akhter S., Islam M.R., Sultana S., Mitra S., Chandran D., Khandaker M.U., Ashraf G.M., Idris A.M. (2022). Exposure of metal toxicity in Alzheimer’s disease: An extensive review. Front. Pharmacol..

[11] Piekut T., Hurła M., Banaszek N., Szejn P., Dorszewska J., Kozubski W., Prendecki M. (2022). Infectious agents and Alzheimer’s disease. J. Integr. Neurosci..

[12] Vigasova D., Nemergut M., Liskova B., Damborsky J. (2021). Multi-pathogen infections and Alzheimer’s disease. Microb. Cell Fact..

[13] Alhazmi H.A., Albratty M. (2022). An update on the novel and approved drugs for Alzheimer disease. Saudi Pharm. J..

[14] Robles A. (2009). Pharmacological Treatment of Alzheimer’s Disease: Is it Progressing Adequately?. Open Neurol. J..

[15] Patwardhan A.G., Belemkar S. (2021). An update on Alzheimer’s disease: Immunotherapeutic agents, stem cell therapy and gene editing. Life Sci..

[16] Temple S. (2023). Advancing cell therapy for neurodegenerative diseases. Cell Stem Cell.

[17] Yoo Y., Neumayer G., Shibuya Y., Mader M.M., Wernig M. (2023). A cell therapy approach to restore microglial Trem2 function in a mouse model of Alzheimer’s disease. Cell Stem Cell.

[18] Qiang X., Li Y., Yang X., Luo L., Xu R., Zheng Y., Cao Z., Tan Z., Deng Y. (2017). DL-3-n-butylphthalide-Edaravone hybrids as novel dual inhibitors of amyloid-β aggregation and monoamine oxidases with high antioxidant potency for Alzheimer’s therapy. Bioorg. Med. Chem. Lett..

[19] Wang Z.G., Sharma A., Feng L., Muresanu D.F., Tian Z.R., Lafuente J.V., Buzoianu A.D., Nozari A., Huang H., Chen L. (2023). Co-administration of dl-3-n-butylphthalide and neprilysin is neuroprotective in Alzheimer disease associated with mild traumatic brain injury. Int. Rev. Neurobiol..

[20] Chen X.Q., Qiu K., Liu H., He Q., Bai J.H., Lu W. (2019). Application and prospects of butylphthalide for the treatment of neurologic diseases. Chin. Med. J..

[21] Li B.Q., Xu L.Z., Li F.Y., Li Y., Zhao Y., Zhang H., Quan M.N., Jia J.P. (2022). CaMKIIα Signaling Is Required for the Neuroprotective Effects of Dl-3-n-Butylphthalide in Alzheimer’s Disease. Mol. Neurobiol..

[22] Wang P., Sun W., Gong J., Han X., Xu C., Chen Y., Yang Y., Luan H., Li S., Li R. (2024). Efficacy and safety of butylphthalide in patients with mild cognitive impairment: A multicentre, randomised, double-blind, placebo-controlled trial (EBMCI study). BMJ Open.

[23] Cheng Z., Li M., Dey R., Chen Y. (2021). Nanomaterials for cancer therapy: Current progress and perspectives. J. Hematol. Oncol..

[24] De Jong W.H., Borm P.J. (2008). Drug delivery and nanoparticles: Applications and hazards. Int. J. Nanomed..

[25] Dong X. (2018). Current Strategies for Brain Drug Delivery. Theranostics.

[26] Xie J., Shen Z., Anraku Y., Kataoka K., Chen X. (2019). Nanomaterial-based blood-brain-barrier (BBB) crossing strategies. Biomaterials.

[27] Zhou X., Smith Q.R., Liu X. (2021). Brain penetrating peptides and peptide-drug conjugates to overcome the blood-brain barrier and target CNS diseases. Wiley Interdiscip. Rev. Nanomed. Nanobiotechnol..

[28] Du C., Feng W., Dai X., Wang J., Geng D., Li X., Chen Y., Zhang J. (2022). Cu^2+^-Chelatable and ROS-Scavenging MXenzyme as NIR-II-Triggered BBlood-Brain Barrier-Crossing Nanocatalyst against Alzheimer’s Disease. Small.

[29] Isfahani A.P., Shamsabadi A.A., Alimohammadi F., Soroush M. (2022). Efficient mercury removal from aqueous solutions using carboxylated Ti_3_C_2_T_x_ MXene. J. Hazard. Mater..

[30] Jin L., Chai L., Yang W., Wang H., Zhang L. (2019). 2D Titanium Carbides (Ti_3_C_2_T_x_) Functionalized by Poly(m-phenylenediamine) for Efficient Adsorption and Reduction of Hexavalent Chromium. Int. J. Environ. Res. Public Health.

[31] Wu Z., Sun D.W., Pu H., Wei Q., Lin X. (2022). Ti_3_C_2_T_x_ MXenes loaded with Au nanoparticle dimers as a surface-enhanced Raman scattering aptasensor for AFB1 detection. Food Chem..

[32] Zhang P., Wang L., Huang Z., Yu J., Li Z., Deng H., Yin T., Yuan L., Gibson J.K., Mei L. (2020). Aryl Diazonium-Assisted Amidoximation of MXene for Boosting Water Stability and Uranyl Sequestration via Electrochemical Sorption. ACS Appl. Mater. Interfaces.

[33] Liu J., Lu W., Lu X., Zhang L., Dong H., Li Y. (2022). Versatile Ti_3_C_2_T_x_ MXene for free-radical scavenging. Nano Res..

[34] Wu C., Xia L., Feng W., Chen Y. (2024). MXene-Mediated Catalytic Redox Reactions for Biomedical Applications. Chempluschem.

[35] Yang Z., Fu X., Ma D., Wang Y., Peng L., Shi J., Sun J., Gan X., Deng Y., Yang W. (2021). Growth Factor-Decorated Ti_3_C_2_ MXene/MoS_2_ 2D Bio-Heterojunctions with Quad-Channel Photonic Disinfection for Effective Regeneration of Bacteria-Invaded Cutaneous Tissue. Small.

[36] Hao S., Han H., Yang Z., Chen M., Jiang Y., Lu G., Dong L., Wen H., Li H., Liu J. (2022). Recent Advancements on Photothermal Conversion and Antibacterial Applications over MXenes-Based Materials. Nano-Micro Lett..

[37] Iravani S., Varma R.S. (2022). MXenes in photomedicine: Advances and prospects. Chem. Commun..

[38] Wu Z., Li C., Li Z., Feng K., Cai M., Zhang D., Wang S., Chu M., Zhang C., Shen J. (2021). Niobium and Titanium Carbides (MXenes) as Superior Photothermal Supports for CO_2_ Photocatalysis. ACS Nano.

[39] Dai C., Chen Y., Jing X., Xiang L., Yang D., Lin H., Liu Z., Han X., Wu R. (2017). 2D Tantalum Carbide (MXenes) Composite Nanosheets for Multiple Imaging-Guided Photothermal Tumor Ablation. ACS Nano.

[40] Deng Y., Zhou Z., Zhang C., Li H., Lan J., Wu J., Wang S. (2023). Enhancing the Ag-loading capacity on Ti_3_C_2_T_x_ sheets as hybrid fillers to form composite coatings with excellent antibacterial properties. RSC Adv..

[41] Dolz D., Morales-García Á., Viñes F., Illas F. (2021). Exfoliation Energy as a Descriptor of MXenes Synthesizability and Surface Chemical Activity. Nanomaterials.

[42] Janica J., Montes-García V., Urban F., Hashemi P., Nia A.S., Feng X., Samorì P., Ciesielski A. (2023). Covalently Functionalized MXenes for Highly Sensitive Humidity Sensors. Small Methods.

[43] Zhao X., Li W.P., Cao Y., Portniagin A., Tang B., Wang S., Liu Q., Yu D.Y.W., Zhong X., Zheng X. (2024). Dual-Atom Co/Ni Electrocatalyst Anchored at the Surface-Modified Ti_3_C_2_T_x_ MXene Enables Efficient Hydrogen and Oxygen Evolution Reactions. ACS Nano.

[44] Zhu H., Dai W., Wang L., Yao C., Wang C., Gu B., Li D., He J. (2022). Electroactive Oxidized Alginate/Gelatin/MXene (Ti_3_C_2_T_x_) Composite Hydrogel with Improved Biocompatibility and Self-Healing Property. Polymers.

[45] Liao M., Cui Q., Hu Y., Xing J., Wu D., Zheng S., Zhao Y., Yu Y., Sun J., Chai R. (2024). Recent advances in the application of MXenes for neural tissue engineering and regeneration. Neural Regen. Res..

[46] Wei H., Gu Y., Li A., Song P., Liu D., Sun F., Ma X., Qian X. (2024). Conductive 3D Ti_3_C_2_T_x_ MXene-Matrigel hydrogels promote proliferation and neuronal differentiation of neural stem cells. Colloids Surf. B Biointerfaces.

[47] Tang Z., Liu Y., Xiang H., Dai X., Huang X., Ju Y., Ni N., Huang R., Gao H., Zhang J. (2023). Bifunctional MXene-Augmented Retinal Progenitor Cell Transplantation for Retinal Degeneration. Adv. Sci..

[48] Li Y., Hu Y., Wei H., Cao W., Qi Y., Zhou S., Zhang P., Li H., Li G.-L., Chai R. (2022). Two-dimensional Ti3C2Tx MXene promotes electrophysiological maturation of neural circuits. J. Nanobiotechnol..

[49] Cai J., Zhang H., Hu Y., Huang Z., Wang Y., Xia Y., Chen X., Guo J., Cheng H., Xia L. (2022). GelMA-MXene hydrogel nerve conduits with microgrooves for spinal cord injury repair. J. Nanobiotechnol..

[50] Dilgimen A.S., Mustafaeva Z., Demchenko M., Kaneko T., Osada Y., Mustafaev M. (2001). Water-soluble covalent conjugates of bovine serum albumin with anionic poly(N-isopropyl-acrylamide) and their immunogenicity. Biomaterials.

[51] Tincu Iurciuc C.E., Daraba O.M., Jérôme C., Popa M., Ochiuz L. (2024). Albumin-Based Hydrogel Films Covalently Cross-Linked with Oxidized Gellan with Encapsulated Curcumin for Biomedical Applications. Polymers.

[52] Gerke C., Zabala Gutierrez I., Méndez-González D., Cruz M.C.I., Mulero F., Jaque D., Rubio-Retama J. (2022). Clickable Albumin Nanoparticles for Pretargeted Drug Delivery toward PD-L1 Overexpressing Tumors in Combination Immunotherapy. Bioconjug. Chem..

[53] Hong Y., Ju Y., Chen W., Liu Y., Zhang M., Zhao H. (2021). Fabrication of PεCL-AuNP-BSA core-shell-corona nanoparticles for flexible spatiotemporal drug delivery and SERS detection. Biomater. Sci..

[54] Huang Y., Hu L., Huang S., Xu W., Wan J., Wang D., Zheng G., Xia Z. (2018). Curcumin-loaded galactosylated BSA nanoparticles as targeted drug delivery carriers inhibit hepatocellular carcinoma cell proliferation and migration. Int. J. Nanomed..

[55] Mazzilli M.R.F., Ambrósio J.A.R., da Silva Godoy D., da Silva Abreu A., Carvalho J.A., Junior M.B., Simioni A.R. (2020). Polyelectrolytic BSA nanoparticles containing silicon dihydroxide phthalocyanine as a promising candidate for drug delivery systems for anticancer photodynamic therapy. J. Biomater. Sci. Polym. Ed..

[56] Wang Y., Chen S., Yang X., Zhang S., Cui C. (2021). Preparation Optimization of Bovine Serum Albumin Nanoparticles and Its Application for siRNA Delivery. Drug Des. Dev. Ther..

[57] Xu L., He X.Y., Liu B.Y., Xu C., Ai S.L., Zhuo R.X., Cheng S.X. (2018). Aptamer-functionalized albumin-based nanoparticles for targeted drug delivery. Colloids Surf. B Biointerfaces.

[58] Choi J.S., Meghani N. (2016). Impact of surface modification in BSA nanoparticles for uptake in cancer cells. Colloids Surf. B Biointerfaces.

[59] Liu Z., Chan L., Ye X., Bai Y., Chen T. (2018). BSA-based Cu_2_Se nanoparticles with multistimuli-responsive drug vehicles for synergistic chemo-photothermal therapy. Colloids Surf. B Biointerfaces.

[60] Qian L., Sun J., Hou C., Yang J., Li Y., Lei D., Yang M., Zhang S. (2017). Immobilization of BSA on ionic liquid functionalized magnetic Fe_3_O_4_ nanoparticles for use in surface imprinting strategy. Talanta.

[61] Hornok V., Amin K.W.K., Kovács A.N., Juhász Á., Katona G., Balogh G.T., Csapó E. (2022). Increased blood-brain barrier permeability of neuroprotective drug by colloidal serum albumin carriers. Colloids Surf. B Biointerfaces.

[62] Hou W., Jiang Y., Xie G., Zhao L., Zhao F., Zhang X., Sun S.K., Yu C., Pan J. (2021). Biocompatible BSA-MnO_2_ nanoparticles for in vivo timely permeability imaging of blood-brain barrier and prediction of hemorrhage transformation in acute ischemic stroke. Nanoscale.

[63] Ma H., Dong Y., Chu Y., Guo Y., Li L. (2022). The mechanisms of ferroptosis and its role in alzheimer’s disease. Front. Mol. Biosci..

[64] Sivaselvam S., Mohankumar A., Thiruppathi G., Sundararaj P., Viswanathan C., Ponpandian N. (2020). Engineering the surface of graphene oxide with bovine serum albumin for improved biocompatibility in Caenorhabditis elegans. Nanoscale Adv..

[65] Ma B.K., Li M., Cheong L.Z., Weng X.C., Shen C., Huang Q. (2020). Enzyme-MXene Nanosheets: Fabrication and Application in Electrochemical Detection of H_2_O_2_. J. Inorg. Mater..

[66] Xu Y., Yang L., Li M., Shu H., Jia N., Gao Y., Shi R., Yang X., Zhang Z., Zhang L. (2024). Anti-osteosarcoma trimodal synergistic therapy using NiFe-LDH and MXene nanocomposite for enhanced biocompatibility and efficacy. Acta Pharm. Sin. B.

[67] Li Z., Zhang H., Han J., Chen Y., Lin H., Yang T. (2018). Surface Nanopore Engineering of 2D MXenes for Targeted and Synergistic Multitherapies of Hepatocellular Carcinoma. Adv. Mater..

[68] Fan L., Lin X., Zhang Y., Zhao W., Chen Y., Wang Y., Wu H., Wang J. (2024). Simultaneous antioxidant and neuroprotective effects of two-dimensional (2D) MXene-loaded isoquercetin for ischemic stroke treatment. J. Mater. Chem. B.

[69] Leuner K., Pantel J., Frey C., Schindowski K., Schulz K., Wegat T., Maurer K., Eckert A., Müller W.E. (2007). Enhanced apoptosis, oxidative stress and mitochondrial dysfunction in lymphocytes as potential biomarkers for Alzheimer’s disease. J. Neural Transm. Suppl..

[70] Leuner K., Schulz K., Schütt T., Pantel J., Prvulovic D., Rhein V., Savaskan E., Czech C., Eckert A., Müller W.E. (2012). Peripheral mitochondrial dysfunction in Alzheimer’s disease: Focus on lymphocytes. Mol. Neurobiol..

[71] She L., Sun J., Xiong L., Li A., Li L., Wu H., Ren J., Wang W., Liang G., Zhao X. (2024). Ginsenoside RK1 improves cognitive impairments and pathological changes in Alzheimer’s disease via stimulation of the AMPK/Nrf2 signaling pathway. Phytomedicine.

[72] Wilkins H.M., Troutwine B.R., Menta B.W., Manley S.J., Strope T.A., Lysaker C.R., Swerdlow R.H. (2022). Mitochondrial Membrane Potential Influences Amyloid-β Protein Precursor Localization and Amyloid-β Secretion. J. Alzheimer’s Dis..

[73] Yang S., Xie Z., Pei T., Zeng Y., Xiong Q., Wei H., Wang Y., Cheng W. (2022). Salidroside attenuates neuronal ferroptosis by activating the Nrf2/HO1 signaling pathway in Aβ(1-42)-induced Alzheimer’s disease mice and glutamate-injured HT22 cells. Chin. Med..

[74] Kurtoglu E., Ugur A., Baltaci A.K., Undar L. (2003). Effect of iron supplementation on oxidative stress and antioxidant status in iron-deficiency anemia. Biol. Trace Elem. Res..

[75] Yan H.-F., Zou T., Tuo Q.-Z., Xu S., Li H., Belaidi A.A., Lei P. (2021). Ferroptosis: Mechanisms and links with diseases. Signal Transduct. Target. Ther..

[76] Cordiano R., Di Gioacchino M., Mangifesta R., Panzera C., Gangemi S., Minciullo P.L. (2023). Malondialdehyde as a Potential Oxidative Stress Marker for Allergy-Oriented Diseases: An Update. Molecules.

[77] Maheshwari S. (2023). Ferroptosis Signaling Pathways: Alzheimer’s Disease. Horm. Metab. Res..

[78] Zhang G., Zhang Y., Shen Y., Wang Y., Zhao M., Sun L. (2021). The Potential Role of Ferroptosis in Alzheimer’s Disease. J. Alzheimer’s Dis..

