# Supplementary material for: Two-Dimensional MXene-Loaded Butylphthalide Enhances the Treatment of Alzheimer’s Disease by Inhibiting Ferroptosis and Oxidative Stress
Source: Pharmaceuticals (Basel). 2026 Sep 1;19(9):1386. doi: 10.3390/ph19091386 (PMC13610566; doi:10.3390/ph19091386)
Supplement: Supplementary file 1 [file pharmaceuticals-19-01386-s001.zip › pharmaceuticals-4348234-supplementary.pdf]

Supplementary Materials

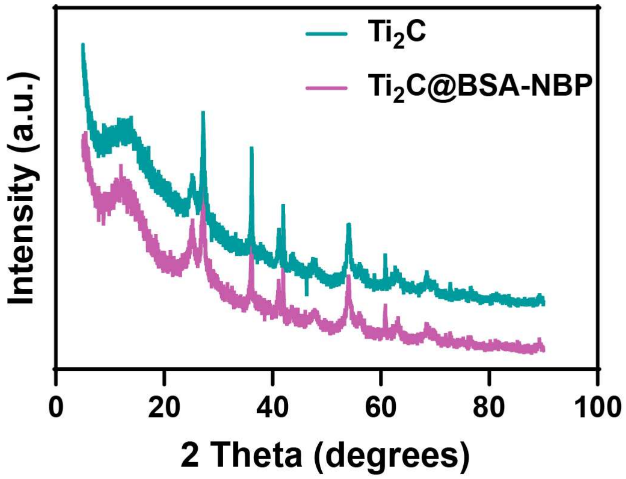

Figure S1. XRD patterns of Ti2C and Ti2C@BSA-NBP. The XRD analysis demonstrates that the crystalline structure of Ti2C was largely retained after grafting with BSA-NBP, indicating that the amidation reaction did not substantially alter the crystallographic structure of Ti2C.

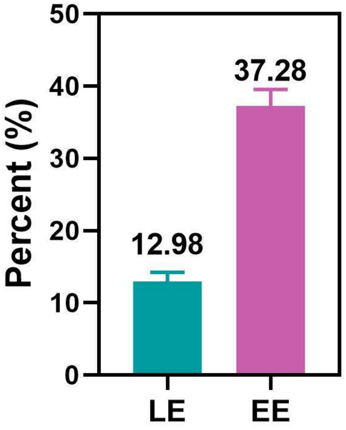

Figure S2. Encapsulation efficiency and loading rate of BSA-NBP nanoparticles. The encapsulation efficiency (EE) and loading efficiency (LE) were determined using the ultrafiltration-differential method. The loading rate was 12.98% and the encapsulation rate was 37.28%.

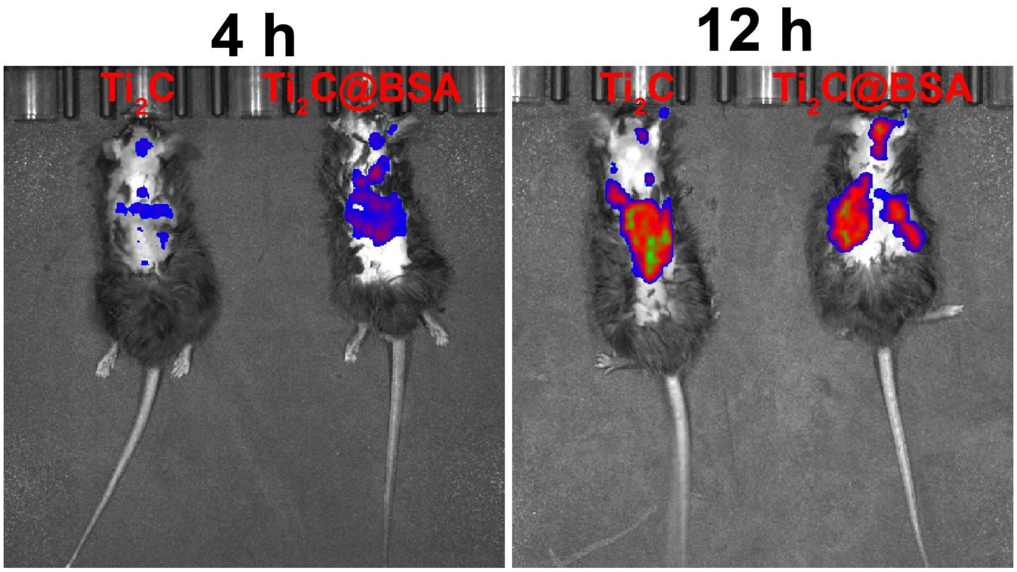

Figure S3. In vivo fluorescence imaging of Cy5-labeled Ti2C and Ti2C@BSA in mice. (A) Whole-body fluorescence imaging at 4 h and 12 h after tail vein injection. (B) Ex vivo organ imaging showing the biodistribution in major organs including brain, heart, liver, spleen, lung, and kidney. Both Ti2C and Ti2C@BSA showed brain accumulation, with Ti2C@BSA exhibiting relatively higher brain enrichment.

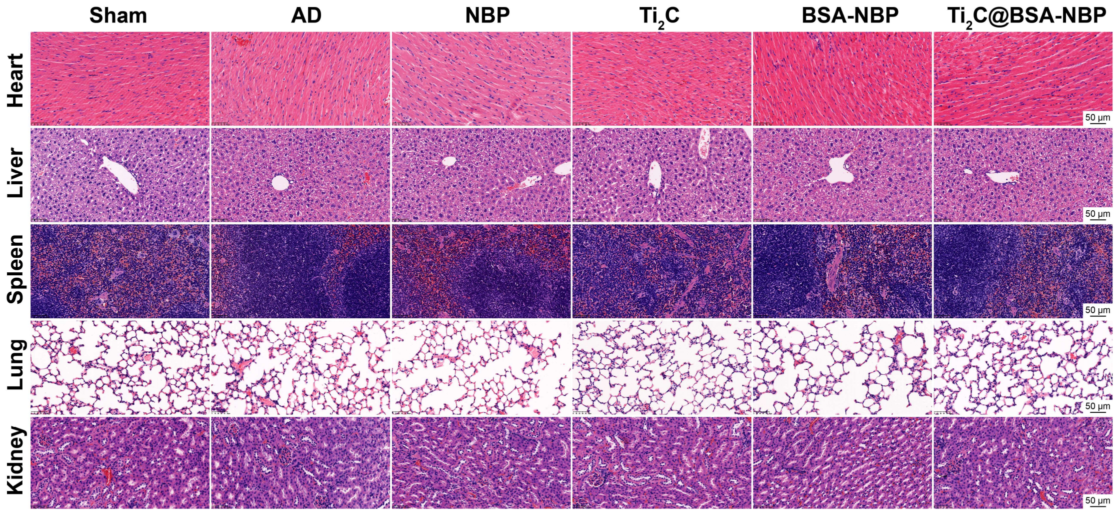

Figure S4. H&E staining of major organs from mice in each group. Representative histological sections of heart, liver, spleen, lung, and kidney showed no obvious pathological changes, indicating that the materials in each group were non-toxic and exhibited good biosafety. Scale bars are indicated on the images.

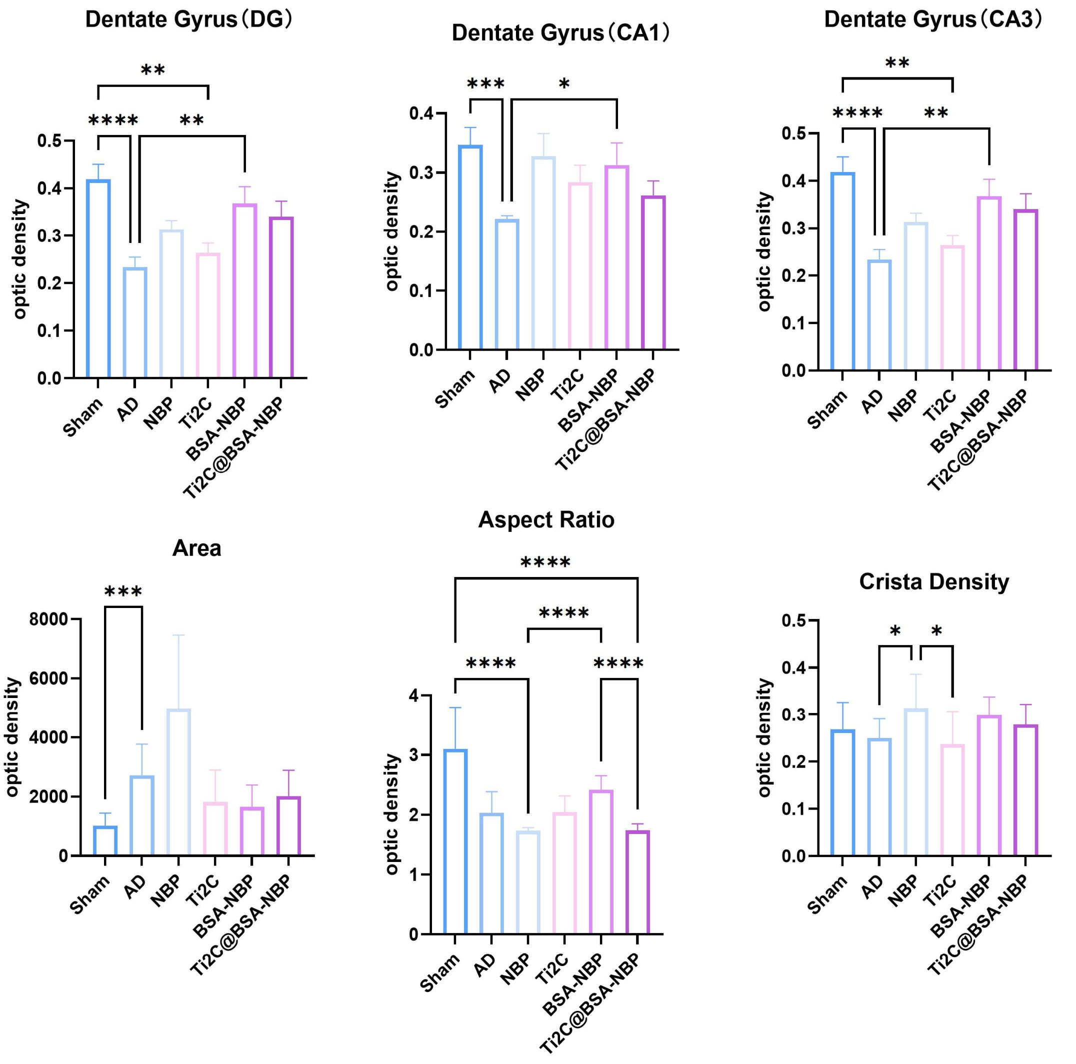

Figure S5. Quantitative analysis of Nissl staining and mitochondrial morphology. (A) Quantitative analysis of Nissl staining optical density in the DG, CA1, and CA3 regions of the hippocampus. (B) Quantitative morphometric analysis of hippocampal mitochondria, including mitochondrial area, aspect ratio, circularity, and cristae density. Data are expressed as mean ± SD (n = 3–6). * $p < 0.05$, ** $p < 0.01$.
